# Role of phospholipase A_2_ receptor 1 antibody level at diagnosis for long-term renal outcome in membranous nephropathy

**DOI:** 10.1371/journal.pone.0221293

**Published:** 2019-09-09

**Authors:** Maida Mahmud, Hans O. Pinnschmidt, Linda Reinhard, Sigrid Harendza, Thorsten Wiech, Rolf A. K. Stahl, Elion Hoxha

**Affiliations:** 1 III. Department of Medicine, University Medical Center Hamburg-Eppendorf, Hamburg, Germany; 2 Institute of Medical Biometry and Epidemiology, University Medical Center Hamburg-Eppendorf, Hamburg, Germany; 3 Division of Nephropathology, Institute of Pathology, University Medical Center Hamburg-Eppendorf, Hamburg, Germany; Istituto Di Ricerche Farmacologiche Mario Negri, ITALY

## Abstract

**Background:**

Membranous nephropathy (MN) is an autoimmune disease induced by circulating antibodies against the podocyte protein phospholipase A_2_ receptor 1 (PLA_2_R1-ab) in 80% of patients and represents the leading cause of nephrotic syndrome in adults. PLA_2_R1-ab levels correlate with disease activity and treatment response. However, their predictive role for long-term renal outcome is not clear.

**Methods:**

The aim of this prospective observational multicenter study was to investigate the predictive role of PLA_2_R1-ab levels at the time of diagnosis for long-term outcome in a cohort of 243 patients with newly diagnosed biopsy-proven PLA_2_R1-associated MN. Statistical analyses included Cox proportional hazard models. The primary study endpoint was defined prior to data collection as doubling of serum creatinine or development of end-stage renal disease.

**Results:**

During the median follow-up time of 48 months, 36 (15%) patients reached the study endpoint. Independent predictors for reaching the study endpoint were baseline PLA_2_R1-ab levels (HR = 1.36, 95%CI 1.11–1.66, p = 0.01), percentage of tubular atrophy and interstitial fibrosis (HR = 1.32, 95%CI 1.03–1.68, p = 0.03), PLA_2_R1-ab relapse during follow-up (HR = 3.22, 95%CI 1.36–7.60, p = 0.01), and relapse of proteinuria (HR = 2.60, 95%CI 1.17–5.79, p = 0.02). Fifty-four (22%) patients received no immunosuppressive treatment during the study, in 41 (76%) of them PLA_2_R1-ab spontaneously disappeared during follow-up, 29 (54%) patients had a complete remission of proteinuria, and 19 (35%) had a partial remission. Patients not treated with immunosuppression were more often females and had lower PLA_2_R1-ab levels, proteinuria, and serum creatinine at baseline compared to patients receiving immunosuppression. However, no conclusion on the efficacy of immunosuppressive therapies can be made, since this was not a randomized controlled study and treatment decisions were not made per-protocol.

**Conclusions:**

PLA_2_R1-ab levels are, in addition to pre-existing renal damage, predictive factors for long-term outcome and should therefore be considered when deciding the treatment of patients with MN.

## Introduction

Membranous nephropathy (MN) is a common cause of nephrotic syndrome in adults. In 80% of the patients the disease is caused by binding of circulating antibodies to the phospholipase A_2_ receptor 1 (PLA_2_R1), which is expressed on the surface of podocytes and is the major target antigen [1]. Detection of PLA_2_R1 antibodies (PLA_2_R1-ab) allows the diagnosis of MN [2–5]. Moreover, PLA_2_R1-ab levels are associated with treatment response, remission of proteinuria, relapse of proteinuria, and recurrence of disease after renal transplantation [4, 6–8]. A number of studies have shown that a decrease of PLA_2_R1-ab precedes clinical remission (i.e. remission of proteinuria) after immunosuppressive treatment [6,9,10]. Furthermore, patients with detectable PLA_2_R1-ab have a higher risk for relapse of proteinuria [11, 12]. Therefore, measurement of PLA_2_R1-ab is helpful for the management of patients with MN [13] but the significance of PLA_2_R1-ab levels for renal endpoints, such as doubling of serum creatinine or development of end-stage renal disease, is not clear because of the retrospective character of the available studies or the short-term follow-up of the patients [14, 15]. While complete remission of proteinuria is considered a surrogate parameter for long-term renal outcome [16], a better understanding of the clinical relevance of PLA_2_R1-ab for long-term prognosis would substantially improve treatment decisions and risk stratification of patients with MN. In order to better define the role of PLA_2_R1-ab levels at the time of diagnosis on long-term renal outcome we conducted a multicenter open prospective observational study in a large cohort of patients with newly diagnosed biopsy-proven PLA_2_R1-associated MN.

## Material and methods

### Patient cohort and study design

Starting from January 2010 all patients with a biopsy-proven diagnosis of MN who fulfilled the study inclusion criteria and provided informed consent to participate in the study were screened for circulating PLA_2_R1-ab. Study enrolment and the first measurement of PLA_2_R1-ab had to be performed within six months of renal biopsy. Treatment with immunosuppressive agents prior to study start was not allowed. Follow-up visits were performed every three months. PLA_2_R1-ab levels, proteinuria, and serum creatinine were measured at every study visit and data were recorded prospectively after each visit. Treatment decisions in enrolled patients were not made per protocol, but by the treating nephrologists, who decided on the therapeutic strategy based on patient characteristics (i.e. proteinuria, nephrotic syndrome, renal function, etc.) and their clinical experience. The primary study endpoint was defined as doubling of serum creatinine in relation to the time of study inclusion or development of end-stage renal disease, whichever occurred earlier. Depletion of PLA_2_R1-ab was defined as PLA_2_R1-ab falling below 14 U/ml. Remission of proteinuria was defined as proteinuria < 3.5 g/24h and at least 50% reduction from the time of study inclusion. Complete remission of proteinuria was defined as proteinuria < 0.5 g/24h. Relapse of PLA_2_R1-ab was defined as PLA_2_R1-ab increasing from < 14 U/ml to a level higher than 20 U/ml. Relapse of proteinuria was defined as proteinuria > 3.5 g/24h and at least doubling of proteinuria compared to the lowest value during the period of remission.

Glomerular disease stages were assessed by electron microscopy according to Ehrenreich and Churg [17]. At study inclusion PLA_2_R1-ab measurement was performed by ELISA, indirect immunofluorescence (IFT) and Western blot, as described before [18]. During follow-up the PLA_2_R1-ab was measured by ELISA [19]. The study was approved by the local ethics committee of the chamber of physicians in Hamburg and conducted in accordance with the ethical principles stated by the Declaration of Helsinki. Informed consent was obtained from all participating patients.

### Statistical analyses

Descriptive analyses of continuous data are presented as median and 1^st^ and 3^rd^ quartile unless stated otherwise. For categorical data, absolute counts and percentages are reported. Mann-Whitney *U* and Kruskal-Wallis tests were employed for comparisons of continuous variables while Fisher’s exact tests were used for group-wise comparisons of categorical variables. Multivariate relationships among variables were explored by nonlinear categorical principal component analysis [20, 21]. A two-dimensional solution was chosen and the component loadings of the variables are graphically presented.

Uni- and multivariate Cox regression analyses were used to assess the effect of independent variables on the time to event for the study endpoint. Time-dependent covariates were computed and used to test the proportional hazards assumption for individual independent variables. Multivariate Cox regression analyses started with an initial model containing only additive terms of independent variables and their corresponding time-dependent terms if either one, the additive, or the time-dependent term, had displayed a significant effect in the univariate analyses. Non-significant terms were stepwise eliminated from the model using a hierarchical backward approach [22]. Results of Cox regression analyses are presented as hazard ratios with corresponding 95% confidence intervals and p-values. Right-skewed covariates were log_2_-transformed to reduce the over-proportional impact of extremely high data values on computations of hazard ratios while at the same time trying to ensure linear relationships to outcomes. These relationships were evaluated via visual examinations of scatter plots of martingale residuals of Cox regressions not containing any covariates in the model versus the respective covariate of interest.

Detailed description of the statistical analyses for the primary study endpoint as well as for the secondary endpoints depletion of PLA_2_R1-ab, relapse of PLA_2_R1-ab, remission of proteinuria, and relapse of proteinuria are included in the supplemental material. Statistical significance was defined as p<0.05. All tests were two-tailed. All statistical analyses were done using SPSS version 25.0 (IBM, Armonk, New York).

### Immunohistochemical staining of renal biopsies for PLA_2_R1

For PLA_2_R1 immunohistochemical analyses slides were deparaffinized, pre-treated in citrate buffer (pH 6.2) for 15 min at 120°C and cooled down in iced water (10 min). After rinsing in 99% ethanol, slides were incubated for 10 min with normal serum (Vector S2000; VectorLaboratories, Burlingame, CA) followed by PLA_2_R1-antibodies (polyclonal antibody from rabbit, 1:3000, HPA 012657, Sigma-Aldrich, St. Louis, MO) overnight at 4°C. The slides were then washed in PBS, incubated with polymer 1 (Zytomed Zytochem-Plus AP Polymer-KitPOLAP), rinsed in PBS and incubated with polymer 2 (Zytomed Zytochem-Plus AP Polymer-Kit POLAP). After washing in PBS, slides were stained in new fuchsin naphthol As-Bi phosphate substrate mixture (30 min) followed by 1 min of nuclear staining in hemalaun (Mayer).

## Results

### Clinical baseline characteristics

A total of 312 consecutive patients with biopsy-proven MN were tested for the presence of PLA_2_R1-ab in the serum. At study inclusion PLA_2_R1-ab were detectable by ELISA and IFT in 222 patients. In addition, 21 patients were tested positive for PLA_2_R1-ab by IFT, but not in the ELISA. These 21 sera were additionally analysed by Western blot and all were positive for PLA_2_R1-ab, confirming the results of the IFT. For 152 patients the renal biopsy was stained for PLA_2_R1 and confirmed the diagnosis of PLA_2_R1-associated MN in all cases. Sixty-nine patients were tested negative for PLA_2_R1-ab by ELISA, IFT, and Western blot and were not included in the study. In five of these patients renal biopsy showed an enhanced staining for PLA_2_R1. Taken together, 243 consecutive patients with the histologic diagnosis of MN and positive PLA_2_R1-ab in the serum were included in this prospective multicenter study. The median follow-up time was 48.0 months (1^st^ to 3^rd^ quartile: 27.0 to 63.0 months), resulting in a cumulative follow-up time of 916.7 patient-years. The clinical baseline characteristics of the study cohort are presented in Table 1. After dividing the study cohort in tertiles according to the PLA_2_R1-ab level at baseline, patients with the highest antibody levels were older, had higher proteinuria and more tubulointerstitial fibrosis, although the absolute difference in the percentage of tubulointerstitial fibrosis between the groups was small. At baseline 63 (26%) patients had an impaired renal function defined as eGFR < 60 ml/min/1.73m^2^ (S1 Table). In addition to having more severe renal damage (serum creatinine, GFR, tubular atrophy and interstitial fibrosis) these patients were also significantly older and had higher proteinuria compared to patients with preserved renal function (eGFR > 60 ml/min/1.73m^2^) at baseline. PLA_2_R1-ab levels were not significantly different between the two groups.

**Table 1:** Clinical baseline characteristics of patients with low (tertile 1), medium (tertile 2), or high (tertile 3) PLA_2_R1-ab level.

		Tertile 1	Tertile 2	Tertile 3	P-value
**Number of Patients**		78	79	79	na
**Age—years** **(median, 1**^**st**^ **- 3**^**rd**^ **quartile)**		47.0 (36.0–61.3)	55.0 (43.0–67.0)	58.0 (46.0–69.0)	0.02
**Male sex (%)**		50 (64%)	60 (76%)	58 (73%)	0.2
**Proteinuria—g/24h** **(median, 1**^**st**^ **- 3**^**rd**^ **quartile)**		6.4 (3.6–8.6)	6.6 (5.0–9.8)	8.4 (5.0–11.8)	0.01
**Serum creatinine—mg/dl** **(median, 1**^**st**^ **- 3**^**rd**^ **quartile)**		0.9 (0.8–1.3)	1.0 (0.8–1.2)	1.0 (0.9–1.3)	0.3
**eGFR, CKD-EPI—mL/min/1.73 m**^**2**^ **(median, 1**^**st**^ **- 3**^**rd**^ **quartile)**		87.0 (62.5–103.4)	87.7 (61.4–103.6)	75.2 (49.2–93.5)	0.1
**PLA**_**2**_**R1-ab level, U/ml** **(median, 1**^**st**^ **- 3**^**rd**^ **quartile)**		36.0 (10.4–57.9)	127.4 (95.3–155.4)	452.0 (274.6–729.4)	<0.001
**Time between renal biopsy and study inclusion–months** **(median, 1**^**st**^ **- 3**^**rd**^ **quartile)**		0.5 (0.2–1.0)	0.5 (0.0–1.0)	0.7 (0.0–1.0)	0.5
**% of tubulointerstitial space with tubular atrophy and interstitial fibrosis (median, 1**^**st**^ **- 3**^**rd**^ **quartile)**		5 (0–20)	5 (0–10)	10 (5–20)	0.01
**Glomerular lesions in renal biopsies (EM)** *	**Stage I (%)**	4 (5%)	5 (7%)	5 (7%)	0.2
	**Stage II (%)**	34 (46%)	48 (66%)	41 (55%)	
	**Stage III (%)**	16 (22%)	12 (16%)	14 (19%)	
	**Stage IV (%)**	20 (27%)	8 (11%)	15 (20%)	

eGFR–estimated GFR according to the CKD-EPI formula; EM–electron microscopy; PLA_2_R1-ab–PLA_2_R1-antibody.

*–data on *Glomerular lesions in renal biopsies (EM)* are available for 222 patients

The component loadings computed by nonlinear categorical principal component analysis to examine relationships among clinical parameters at baseline reveal positive correlations among serum creatinine levels at baseline, extent of tubular atrophy and interstitial fibrosis, and age of patients while these clinical parameters show a strong negative correlation with eGFR. PLA_2_R1-ab appears unrelated to any of these clinical baseline parameters (S1 Fig).

### Clinical variables associated with the primary study endpoint

**Fig 1:**
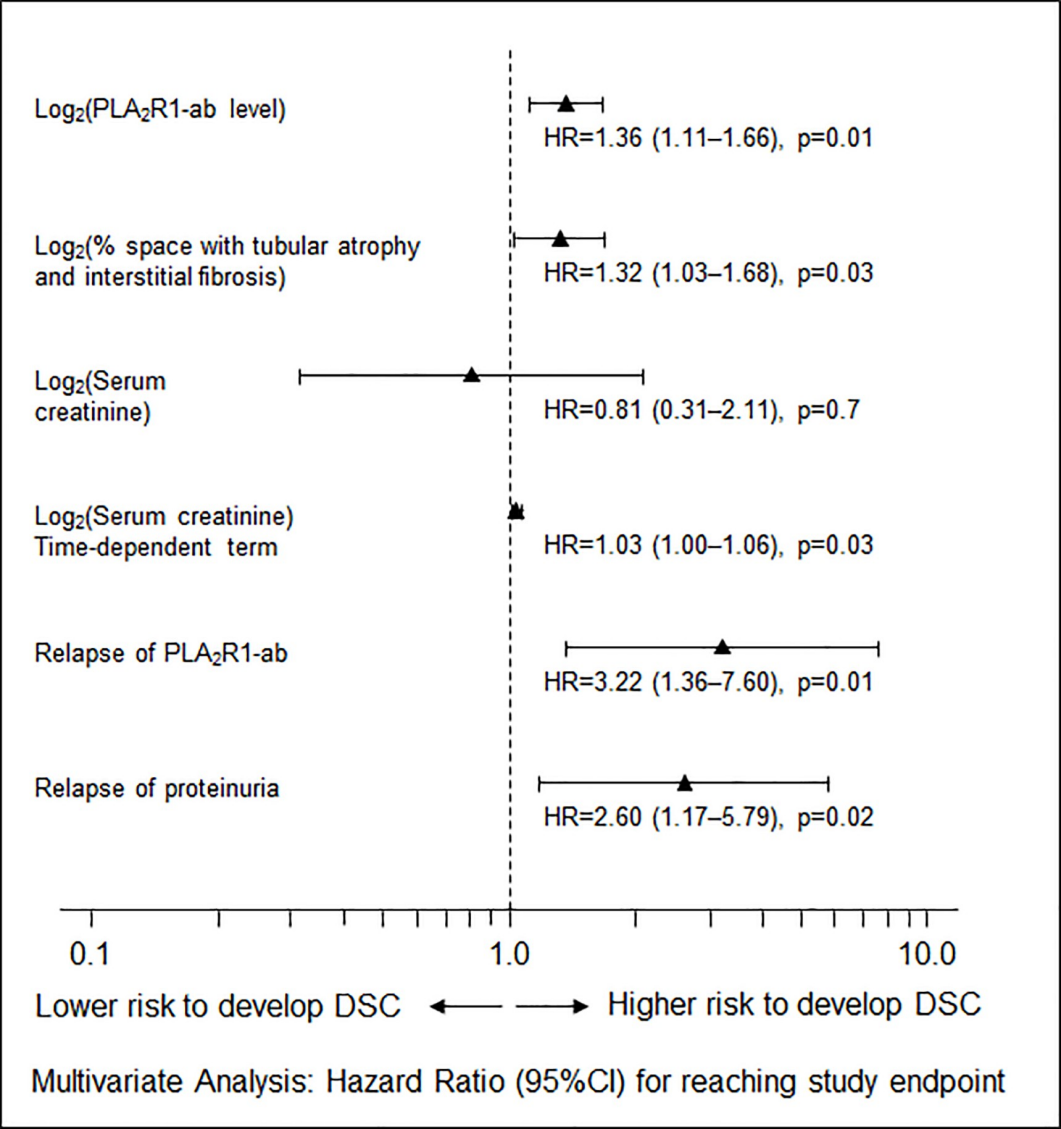
Multivariate Cox regression analysis. PLA_2_R1-ab levels and the extent of tubular atrophy and interstitial fibrosis were the only variables identified as risk factors at baseline for reaching the study endpoint. Both variables were transformed to their binary logarithm prior to using them in these analyses. Relapse of PLA_2_R1-ab and proteinuria were the follow-up parameters found to be risk factors for reaching the study endpoint. We adjusted the analysis of all variables for time-varying effects during follow-up and found a significant time-dependent change of the variable effect for serum creatinine. However, the effect of serum creatinine for the study endpoint was not significant. 95%CI: 95% Confidence Interval; HR: hazard ratio; PLA_2_R1-ab: PLA_2_R1-antibody; DSC: doubling of serum creatinine.

Thirty-six (15%) patients reached the study endpoint defined as doubling of serum creatinine or development of end-stage renal disease. These patients reached the study endpoint after a median follow-up time of 18.0 months (1^st^ to 3^rd^ quartile: 12.0 to 42.0 months), 14 (5.8%) of these patients developed end-stage renal disease. Results of univariate Cox regression analyses testing all independent variables and their time-dependent terms individually are provided in S2 Table. In the multivariate Cox regression analysis higher PLA_2_R1-ab levels at baseline significantly increased the risk for reaching the study endpoint (log_2_[PLA_2_R1-ab levels]: HR = 1.36, 95%CI 1.11–1.66, p = 0.01, Fig 1). Of all other baseline clinical parameters, only the percentage of tubular atrophy and interstitial fibrosis was a statistically significant risk factor (log_2_[tubular atrophy and interstitial fibrosis] HR = 1.32, 95%CI 1.03–1.68, p = 0.03). After analyzing all variables for time-varying effects during follow-up a significant time-dependent change of the variable effect was found for serum creatinine, showing that the variable effect of serum creatinine for the study endpoint significantly increases during the follow-up time. In this multivariate Cox regression analysis we also included parameters associated with disease progression and treatment response during follow-up and found that relapse of PLA_2_R1-ab during follow-up and a relapse of proteinuria significantly increased the risk for reaching the study endpoint (HR = 3.22, 95%CI 1.36–7.60, p = 0.01 and HR = 2.60 95%CI 1.17–5.79, p = 0.02, respectively).

Patients who reached the study endpoint had significantly higher PLA_2_R1-ab, higher serum creatinine levels, lower eGFR, more extended tubular atrophy and interstitial fibrosis at baseline, while during follow-up they more often failed to deplete PLA_2_R1-ab and had significantly less often a complete remission of proteinuria compared to patients who did not reach the study endpoint (Table 2).

**Table 2:** Clinical characteristics of patients who reached the primary study endpoint compared to patients who did not.

			Complete cohort	No DSC	DSC	P-value
**Number of Patients**			243	207	36	na
**Age—years** **(median, 1**^**st**^ **- 3**^**rd**^ **quartile)**			55.0 (43.0–65.5)	54.0 (42.0–64.0)	59.5 (50.3–69.0)	0.07
**Male sex (%)**			171 (70%)	145 (70%)	26 (72%)	0.8
**Proteinuria—g/24h** **(median, 1**^**st**^ **- 3**^**rd**^ **quartile)**			7.0 (4.8–10.6)	6.8 (4.7–10.5)	7.8 (4.9–10.6)	0.8
**Serum creatinine—mg/dl** **(median, 1**^**st**^ **- 3**^**rd**^ **quartile)**			1.0 (0.8–1.3)	1.0 (0.8–1.2)	1.2 (0.9–1.9)	<0.001
**eGFR, CKD-EPI—mL/min/1.73 m**^**2**^ **(median, 1**^**st**^ **- 3**^**rd**^ **quartile)**			82.7 (58.0–100.5)	84.7 (62.6–102.1)	53.7 (31.3–95.4)	0.01
**PLA**_**2**_**R1-ab level, U/ml** **(median, 1**^**st**^ **- 3**^**rd**^ **quartile)**			127.7 (59.3–276.6)	104.9 (53.0–235.5)	315.2 (132.3–749.1)	<0.001
**Time between renal biopsy and study inclusion–months** **(median, 1**^**st**^ **- 3**^**rd**^ **quartile)**			0.5 (0.0–1.0)	0.5 (0.0–1.0)	0.5 (0.2–1.0)	0.9
**% of tubulointerstitial space with tubular atrophy and interstitial fibrosis (median, 1**^**st**^ **- 3**^**rd**^ **quartile)**			5 (0–20)	5 (0–15)	20 (10–50)	<0.001
**Glomerular lesions in renal biopsies (EM)** *		**Stage I (%)**	14 (6%)	14 (7%)	0 (0%)	0.1
		**Stage II (%)**	123 (55%)	102 (54%)	21 (62%)	0.5
		**Stage III (%)**	42 (19%)	39 (21%)	3 (9%)	0.2
		**Stage IV (%)**	43 (19%)	33 (18%)	10 (29%)	0.1
**Immunosuppressive treatment during follow-up (%)**			189 (78%)	158 (76%)	31 (86%)	0.3
**PLA**_**2**_**R1-ab persistent throughout the follow-up (%)**			49 (20%)	34 (16%)	15 (42%)	0.001
**Relapse of PLA**_**2**_**R1-ab during follow-up (%)**			72 (30%)	60 (29%)	12 (33%)	0.7
**Remission of proteinuria**	**CR (%)**		135 (56%)	123 (59%)	12 (33%)	0.01
	**PR (%)**		81 (33%)	67 (32%)	14 (39%)	0.4

DSC–doubling of serum creatinine; eGFR–estimated GFR according to the CKD-EPI formula; EM–electron microscopy; PLA_2_R1-ab–PLA_2_R1-antibody; CR–complete remission; PR–partial remission

*–data on *Glomerular lesions in renal biopsies (EM)* are available for 222 patients

### Secondary study endpoints

Since relapse of PLA_2_R1-ab and proteinuria during follow-up significantly increased the risk for reaching the primary study endpoint, we analysed in a next step which clinical parameters might influence the outcome of PLA_2_R1-ab and proteinuria during follow-up (supplemental methods). We first performed a univariate Cox regression analysis to identify clinical variables potentially linked to depletion of PLA_2_R1-ab (S3 Table), relapse of PLA_2_R1-ab (S4 Table), remission of proteinuria (S5 Table), and relapse of proteinuria (S6 Table). In a second step we performed multivariate Cox regression analyses and identified PLA_2_R1-ab levels at baseline to be a significant predictor of depletion of PLA_2_R1-ab, remission of proteinuria, and relapse of proteinuria during follow-up (log_2_[PLA_2_R1-ab levels]: HR = 0.71, 95%CI 0.63–0.79, p<0.001; HR = 0.92, 95%CI 0.86–0.99, p = 0.02; and HR = 1.15, 95%CI 1.03–1.28, p = 0.01, respectively; S2 and S3 Figs). PLA_2_R1-ab levels at baseline were also predictive for relapse of PLA_2_R1-ab during follow-up in the univariate analysis, however, in the multivariate analysis this association was not statistically significant (log_2_[PLA_2_R1-ab levels]: HR 1.12, 95%CI 0.99–1.26, p = 0.08). The only other parameter associated with depletion of PLA_2_R1-ab in the multivariate analysis was use of immunosuppressive treatment (HR 4.15, 95%CI 2.84–6.06, p<0.001; S2 Fig). We also found a significant time-dependent change of the variable effect for PLA_2_R1-ab and age, showing that the effect of these variables for the endpoint significantly changes during the follow-up time.

In addition to the PLA_2_R1-ab levels at baseline, depletion of PLA_2_R1-ab, and proteinuria at baseline were also significant risk factors for remission of proteinuria (HR = 2.56, 95%CI 1.81–3.61, p<0.001 and log_2_[proteinuria]: HR = 0.65, 95%CI 0.50–0.83, p<0.001, respectively; S3A Fig). Moreover, proteinuria and serum creatinine showed a significant time-dependent change of the variable effect during the follow-up time. In addition to PLA_2_R1-ab levels, serum creatinine at baseline, relapse of PLA_2_R1-ab, and partial remission of proteinuria compared to complete remission significantly increased the risk for a relapse of proteinuria (log_2_[serum creatinine]: HR = 1.77, 95%CI 1.24–2.52, p = 0.01; HR = 3.06, 95%CI 1.81–5.16, p<0.001; and HR = 10.00, 95%CI 5.97–16.76, p<0.001, respectively; S3B Fig).

### Patients treated with immunosuppression or supportive care only

**Fig 2:**
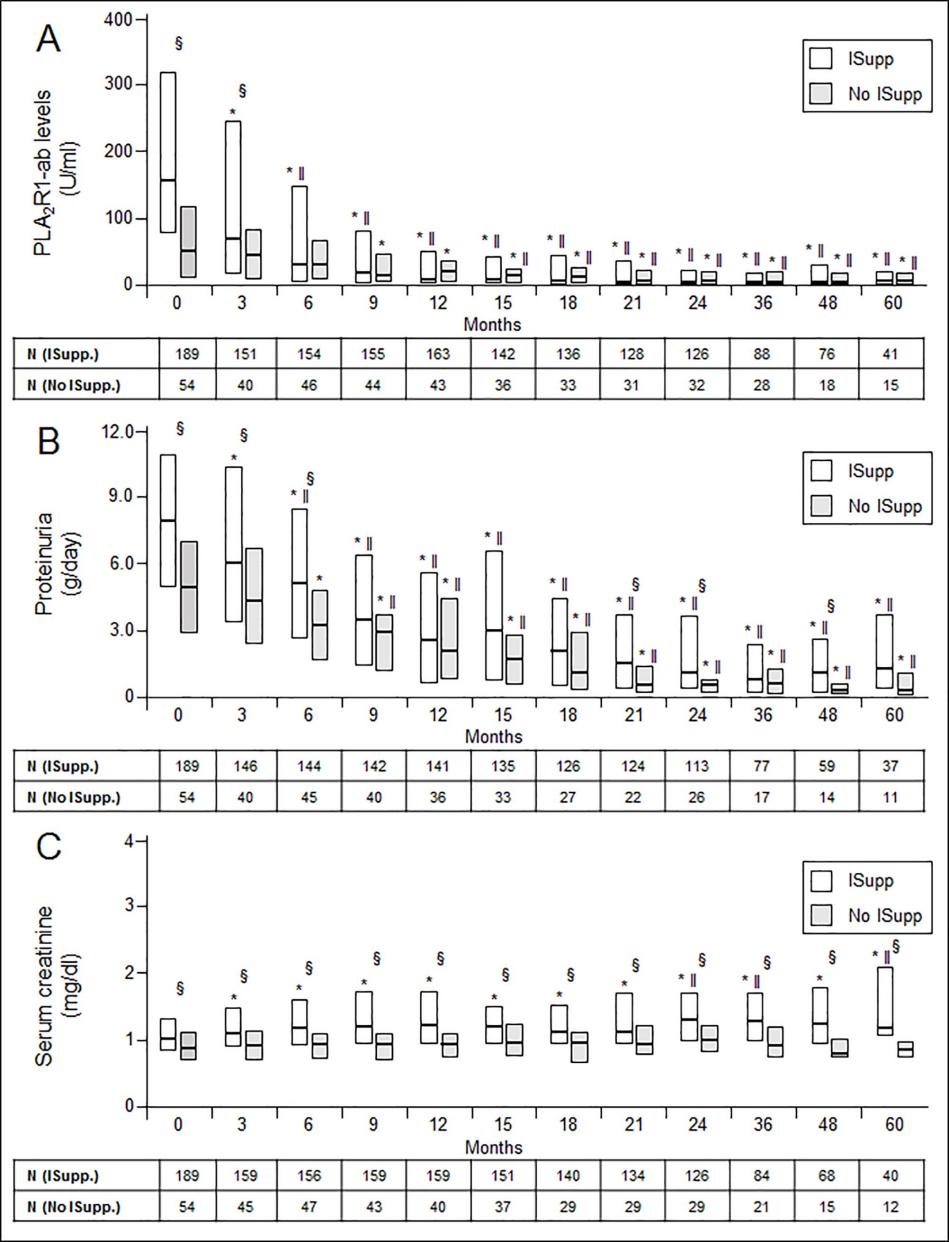
PLA_2_R1-ab levels, proteinuria, and serum creatinine in patients treated with immunosuppression, or supportive medication only. PLA_2_R1-ab levels (A) and proteinuria (B) significantly decreased in patients treated with immunosuppression after three months (*) and between three and six months (ǁ). Serum creatinine increased in patients treated with immunosuppression after three months, but remained stable thereafter (C). At baseline patients treated with immunosuppressants had significantly higher PLA_2_R1-ab levels, proteinuria, and serum creatinine compared to patients who received no immunosuppression (§). The statistical significance was lost at six months for PLA_2_R1-ab, at nine months for proteinuria, but persisted for serum creatinine. In patients who received supportive treatment only the decrease of PLA_2_R1-ab levels and proteinuria reached statistical significance after nine months and six months, respectively. Time “0” shows data at the time of the first PLA_2_R1-ab measurement. ISupp.: Patients treated with immunosuppression. No ISupp.: patients treated with supportive medication only. “*” shows a statistically significant difference between the single time point and the start of the study (p<0.05). “ǁ” shows a statistically significant difference between the single time point and the time 3 months after study start (p<0.05). “§” shows a statistically significant difference between patients treated with immunosuppressive treatment and those who received supportive treatment only (p<0.05). PLA_2_R1-ab: PLA_2_R1 antibody.

During the study, 189 (78%) patients were treated with immunosuppressive agents in addition to supportive medication, while 54 (22%) patients received supportive medication only (S7 Table). Patients treated with immunosuppression were significantly more often male (p = 0.03), had higher PLA_2_R1-ab levels (p<0.001), higher proteinuria (p<0.001), higher serum creatinine at baseline (p = 0.01), and more often a relapse of PLA_2_R1-ab during follow-up (p = 0.04) compared to the 54 patients who were treated with supportive care only. However, the differences in PLA_2_R1-ab levels and proteinuria between patients treated with immunosuppression and patients receiving only supportive medication diminished during follow-up, most probably as an effect of the started immunosuppressive therapy, and were no longer significant after six months and nine months, respectively (Fig 2). Patients treated with immunosuppressants had higher serum creatinine levels compared to patients treated with supportive medication only and this difference persisted throughout the study follow-up.

### Natural course and outcome of PLA_2_R1-associated MN

We further analysed the 54 patients who were treated with supportive care only, in order to better understand the natural course of disease, when no immunosuppressive treatment is given. Forty-one (76%) of these patients spontaneously reduced their PLA_2_R1-ab levels during follow-up (S8 Table). The only statistically significant difference between the two patient cohorts at baseline were the PLA_2_R1-ab levels. Patients with spontaneous reduction of PLA_2_R1-ab levels had significantly more often a remission of proteinuria (98% of patients, 71% complete remission and 27% partial remission) compared to patients with persistent PLA_2_R1-ab (62% of patients, 0% complete remission and 62% partial remission). Patients with spontaneous reduction of PLA_2_R1-ab reached less often the study endpoint, however, this difference did not reach statistical significance (5% versus 23%, p = 0.08).

### Effect of individual immunosuppressive treatments

The initial immunosuppressive agent chosen in most patients was cyclosporine A (81 patients, in 66 of them combined with steroids), followed by oral cyclophosphamide (39 patients, in 38 of them combined with steroids), intravenous (iv) cyclophosphamide (35 patients, in 28 of them combined with steroids), and rituximab (19 patients, in six of them combined with steroids). Immunosuppressive treatment was started at 3.0 months (1^st^ to 3^rd^ quartile: 0.0 to 6.0 months) after inclusion in the study. Proteinuria, renal function, and PLA_2_R1-ab levels were not significantly different between study inclusion and start of treatment (S9 and S10 Tables).

**Fig 3:**
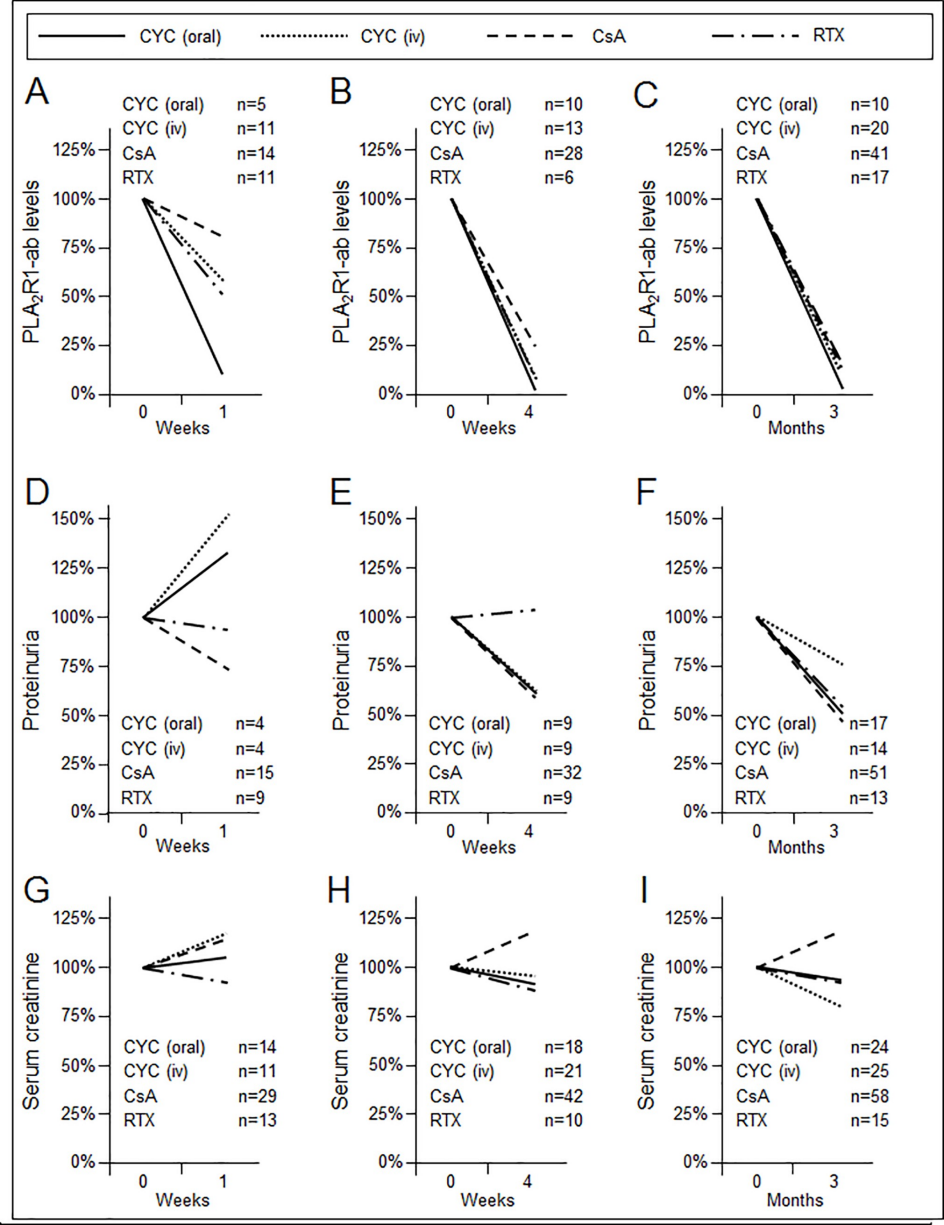
Changes in PLA_2_R1-ab levels, proteinuria, and serum creatinine following immunosuppressive therapy. Data are presented as the relation of PLA_2_R1-ab (Fig 3A, 3B and 3C), proteinuria (Fig 3D, 3E and 3F) and serum creatinine (Fig 3G, 3H and 3I) at the given time point (one week: Fig 3A, 3D, 3G; four weeks: Fig 3B, 3E, 3H; three months: Fig 3C, 3F, 3I) to the time of start of immunosuppression (Time 0). Data are presented for patients treated with oral cyclophosphamide, iv cyclophosphamide, cyclosporine A, or rituximab. Time “0” shows data at the time of the start of immunosuppression. CYC: cyclophosphamide; CsA: cyclosporine A; RTX: rituximab; iv: intravenous; PLA_2_R1-ab: PLA_2_R1 antibody.

In some of the patients, data on PLA_2_R1-ab, proteinuria, and serum creatinine were collected at a short time after start of immunosuppressive treatment, namely one week and four weeks (Fig 3). We noticed a decrease of PLA_2_R1-ab levels already after one week in all treatment groups, which was most pronounced in patients treated with oral cyclophosphamide (86%), followed by rituximab (47%), iv cyclophosphamide (40%), and cyclosporine A (18%) (Fig 3A). After four weeks and three months the PLA_2_R1-ab levels had decreased in all groups by 74%– 94% and 85%– 97%, respectively (Fig 3B and 3C). After one week, proteinuria was only reduced in patients treated with cyclosporine A (26%), and rituximab (5%), but not in patients treated with cyclophosphamide (Fig 3D). After four weeks, proteinuria fell by 40% in all groups, except for patients treated with rituximab (Fig 3E). Only after three months a decrease of proteinuria by 25%– 54% was observed in all groups (Fig 3F). Serum creatinine decreased after one week in patients treated with rituximab (7%), but increased by 5%, 14%, and 17% in patients treated with oral cyclophosphamide, cyclosporine A, and iv cyclophosphamide, respectively (Fig 3G). After four weeks, serum creatinine fell by 6%– 12% in all groups, except patients treated with cyclosporine A, in whom serum creatinine increased by 18% (Fig 3H). This pattern was also observed at three months, when a decrease of serum creatinine by 6%– 19% was observed in all groups, except patients treated with cyclosporine A, in whom serum creatinine increased by 18% (Fig 3I). In 43 patients treated with cyclosporine A, we were able to analyse data on serum creatinine at the time when cyclosporine A was stopped and within 3 months after cessation of treatment with cyclosporine A. Within this short time period (median 2.0 months, 1^st^ to 3^rd^ quartile: 1.0 to 3.0 months) serum creatinine decreased by 9.1% in these patients.

In every treatment group in almost half of the patients the initial immunosuppressive treatment was changed from one medication to another (42% of patients treated with rituximab, 46% of patients treated with oral cyclophosphamide, 52% of patients treated with cyclosporine A and 57% of patients treated with iv cyclophosphamide). However, no statistically significant differences were observed between the baseline clinical characteristics and outcome parameters of patients who needed a second line immunosuppressive treatment and patients who did not need a second line immunosuppressive treatment (S11 Table).

## Discussion

The identification of PLA_2_R1 as the major target antigen in MN has led to significant improvements in the diagnosis and treatment of MN [1, 13]. The role of PLA_2_R1-ab as a biomarker for disease activity and treatment response has been shown in several studies, but almost all of these studies had a retrospective design and were built on short-term surrogate endpoints like proteinuria [4, 6, 8, 11, 12, 23].The identification of biomarkers for hard renal endpoints in patients with MN is important not only for treatment strategies, but also for the design of future therapeutic studies. Some studies analysing the role of PLA_2_R1-ab for the outcome of renal function only had a retrospective design, short term follow-up, and the renal endpoint was defined as a rather small increase in renal retention parameters [14, 15].

This is the first prospective study to investigate the predictive role of PLA_2_R1-ab for long-term clinical outcome, i.e. doubling of serum creatinine in a large cohort of patients with newly diagnosed PLA_2_R1-associated MN. High PLA_2_R1-ab levels at baseline were found to be a significant risk factor for doubling of serum creatinine. The hazard ratio of 1.36 per 2-fold-increase of PLA_2_R1-ab levels underscores their relevance for the clinical outcome of patients with MN. At baseline, only parameters indicating renal damage, e.g. tubular atrophy and interstitial fibrosis, were found to be a risk factor for reaching the study endpoint in addition to the PLA_2_R1-ab level. The IFT was more sensitive than the ELISA for detection of PLA_2_R1-ab, as we have shown earlier [2, 6]. At the same time, PLA_2_R1-ab levels were not closely related to any of the baseline clinical characteristics as shown in the nonlinear categorical principal component analysis which indicated that serum creatinine, tubular atrophy and interstitial fibrosis, and age of patients were sharing close relations to a common dimension. Our data also confirmed the relevance of clinical parameters associated with disease progression and treatment response during follow-up. Relapse of PLA_2_R1-ab and proteinuria during follow-up were significant factors for loss of renal function. Importantly, high PLA_2_R1-ab levels at baseline were identified as a risk factor for almost every parameter of disease activity during follow-up (depletion of PLA_2_R1-ab, remission of proteinuria, and relapse of proteinuria), in addition to the study endpoint. Moreover, depletion of PLA_2_R1-ab was predictive for remission of proteinuria, while relapse of PLA_2_R1-ab was predictive for relapse of proteinuria. As has been shown before [16], patients with a partial remission of proteinuria had a much higher risk for relapse of proteinuria compared to patients with a complete remission of proteinuria (HR = 10.0).

Additionally, we had the chance to study the natural course of PLA_2_R1-associated MN, since 22% of the patients were treated with supportive medication only. Since these patients had lower PLA_2_R1-ab levels, proteinuria, and serum creatinine at baseline compared to patients treated with immunosuppression, they might represent a cohort of patients with low disease activity. In most of these patients PLA_2_R1-ab spontaneously disappeared during follow-up. This is an important observation, since it suggests that in a considerable number of patients unknown mechanisms lead to spontaneous disappearance of PLA_2_R1-ab from the circulation. A better pathophysiologic characterisation of the mechanisms responsible for this phenomenon might lead to new treatment options for MN.

In a part of the study cohort we analysed the development of PLA_2_R1-ab, proteinuria, and serum creatinine within a very short time after start of immunosuppression. As we had observed in single cases of MN before [24], we found a very rapid decline of PLA_2_R1-ab upon immunosuppressive treatment, in some patients within a week. After four weeks, PLA_2_R1-ab levels had declined by almost 80%, while proteinuria declined at a slower rate by about 40% at four weeks, which also has been observed by others [9, 10]. An intriguing finding in our cohort was that four weeks and three months after start of immunosuppressive treatment serum creatinine declined in all treatment groups, except in patients treated with cyclosporine A, in whom serum creatinine increased by 18% at both time points. The very short latency of this effect and the fact that within three months after cessation of cyclosporine A serum creatinine decreased by 9.1% suggest that these findings represent a hemodynamic effect of cyclosporine A. Taken together, we found no significant difference in the effect of the different immunosuppressants on PLA_2_R1-ab or proteinuria in our study cohort since in every treatment group immunosuppressive treatment had to be changed because of treatment failure or adverse effects in 42% - 57% of the patients. The identification of biomarkers, which may allow a prognosis on the efficacy of a specific immunosuppressive treatment in individual patients would be a significant improvement for the management of patients with MN.

Our study has a number of limitations. This was not a randomized controlled study, therefore no final conclusion can be made on the efficacy of the individual immunosuppressive treatments, especially concerning their long-term efficacy on renal function. Moreover, patients treated only with supportive medication might represent a subclass of patients with low disease activity, rather than the random MN patient in the daily clinical routine. Nonetheless, considering the good outcome of disease in these patients, their identification is an unmet need in clinical routine.

PLA_2_R1-ab at baseline are an important risk factor for the long-term renal outcome of patients with MN and should therefore be embedded in the decision making and treatment management of these patients. Spontaneous disappearance of PLA_2_R1-ab from the circulation is not uncommon and associated with a very good outcome. A better understanding of the immunologic mechanisms leading to this finding might lead to new treatment options for MN.

## Supporting information

S1 Fig Component loading vectors of all clinical baseline variables. The cosine of the angle between the component loading vectors of the variables represents the correlation among the respective variables in the shown two-dimensional solution. An angle close to 0° indicates high positive correlation, an angle close to 180° indicates negative correlation, angles around 90° indicate no correlation. The length of a vector indicates the importance of the respective variable for the two-dimensional solution. eGFR: estimated GFR based on the CKD-EPI formula. PLA_2_R1-ab: PLA_2_R1 antibody.
(DOCX)

S2 Fig Multivariate Cox regression analysis for depletion and relapse of PLA_2_R1-ab. A: PLA_2_R1-ab levels at baseline and use of immunosuppressive treatment were the only variables significantly associated with depletion of PLA_2_R1-ab during follow-up. The variable PLA_2_R1-ab level was transformed to its binary logarithm for this analysis. We adjusted the analysis for time-varying effects during follow-up and found a significant time-dependent change of the variable effect for both PLA_2_R1-ab levels and age. However, the effect of age for the endpoint was not significant. B: Use of immunosuppression was the only variable identified as a significant risk factor for relapse of PLA_2_R1-ab. 95%CI: 95% Confidence Interval; HR: hazard ratio; PLA_2_R1-ab: PLA_2_R1-antibody.
(DOCX)

S3 Fig Multivariate Cox regression analysis for remission and relapse of proteinuria. A: PLA_2_R1-ab levels, proteinuria at baseline and depletion of PLA_2_R1-ab levels during follow-up were risk factors for remission of proteinuria. We adjusted the analysis for time-varying effects during follow-up and found a significant time-dependent change of the variable effect for both proteinuria and serum creatinine. However, the effect of serum creatinine for the study end point was not significant. B: PLA_2_R1-ab levels, serum creatinine at baseline, relapse of PLA_2_R1-ab levels and partial remission of proteinuria (compared to complete remission) were identified as significant risk factors for relapse of proteinuria. The variables PLA_2_R1-ab level, proteinuria, and serum creatinine were transformed to their binary logarithm prior to using them in the Cox regression analyses. 95% CI: 95% confidence interval; HR: hazard ratio; PLA_2_R1-ab: PLA_2_R1-antibody.
(DOCX)

S1 Table Clinical baseline characteristics and outcomes of patients with eGFR below or higher than 60 mL/min/1.73 m^2^ at baseline. eGFR–estimated GFR according to the CKD-EPI formula; PLA_2_R1-ab–PLA_2_R1-antibody.
(DOCX)

S2 Table Univariate Cox regression analysis to identify clinical parameters predictive for the study endpoint. In the analyses of independent variables measured at baseline we adjusted the analysis for potential time-varying effects during follow-up, while for independent variables representing events measured during follow-up we adjusted the effect of the variable for the time when its event occurred. This table presents results of each variable both with and without adjusting for these time-dependent effects. Un-adjusted analyses consider only a main effect term for each variable. Analyses of baseline variables which were adjusted for time-varying effects consider a main effect term (reflecting the initial effect of the variable) and a time-dependent term (reflecting the change of the variable effect during time). Analyses adjusted for time-varying effects of event variables measured during follow-up consider only a time-dependent term (reflecting the effect of the variable from the time when its event occurs). 95% Conf. Interval: 95% Confidence Interval; PLA_2_R1-ab: PLA_2_R1-antibody; Time-dep.: time-dependent; CR: complete remission; PR: partial remission.
(DOCX)

S3 Table Univariate Cox regression analysis for depletion of PLA_2_R1-ab. In the analyses of independent variables measured at baseline we adjusted the analysis for potential time-varying effects during follow-up. In this table we present results of each variable both with, and without adjusting for these time-dependent effects. Unadjusted analyses consider only a main effect term for each variable. Analyses of baseline variables which were adjusted for time-varying effects consider a main effect term (reflecting the initial effect of the variable) and a time-dependent term (reflecting the change of the variable effect during time). In the analysis of “use of immunosuppressive treatment” when adjusting for time-varying effects we consider only a time-dependent term (reflecting the effect of the variable from the time when the event occurs–immunosuppression is started). 95% Conf. Interval: 95% Confidence Interval; PLA_2_R1-ab: PLA_2_R1-antibody; Time-dep.: time-dependent.
(DOCX)

S4 Table Univariate Cox regression analysis for relapse of PLA_2_R1-ab. In the analyses of independent variables measured at baseline we adjusted the analysis for potential time-varying effects during follow-up. In this table we present results of each variable both with, and without adjusting for these time-dependent effects. Unadjusted analyses consider only a main effect term for each variable. Analyses of baseline variables which were adjusted for time-varying effects consider a main effect term (reflecting the initial effect of the variable) and a time-dependent term (reflecting the change of the variable effect during time). In the analysis of “use of immunosuppressive treatment” when adjusting for time-varying effects we consider only a time-dependent term (reflecting the effect of the variable from the time when the event occurs–immunosuppression is started). 95% Conf. Interval: 95% Confidence Interval; PLA_2_R1-ab: PLA_2_R1-antibody; Time-dep.: time-dependent.
(DOCX)

S5 Table Univariate Cox regression analysis for remission of proteinuria. In the analyses of independent variables measured at baseline we adjusted the analysis for potential time-varying effects during follow-up. In this table we present results of each variable both with, and without adjusting for these time-dependent effects. Unadjusted analyses consider only a main effect term for each variable. Analyses of baseline variables which were adjusted for time-varying effects consider a main effect term (reflecting the initial effect of the variable) and a time-dependent term (reflecting the change of the variable effect during time). Analyses adjusted for time-varying effects of event variables measured during follow-up consider only a time-dependent term (reflecting the effect of the variable from the time when its event occurs). 95% Conf. Interval: 95% Confidence Interval; PLA_2_R1-ab: PLA_2_R1-antibody; Time-dep.: time-dependent.
(DOCX)

S6 Table Univariate Cox regression analysis for relapse of proteinuria. We adjusted in the analyses of independent variables measured at baseline for potential time-varying effects during follow-up. In this table we present results of each variable both with, and without adjusting for these time-dependent effects. Unadjusted analyses consider only a main effect term for each variable. Analyses of baseline variables which were adjusted for time-varying effects consider a main effect term (reflecting the initial effect of the variable) and a time-dependent term (reflecting the change of the variable effect during time). Analyses adjusted for time-varying effects of event variables measured during follow-up consider only a time-dependent term (reflecting the effect of the variable from the time when its event occurs). 95% Conf. Interval: 95% Confidence Interval; PLA_2_R1-ab: PLA_2_R1-antibody; Time-dep.: time-dependent; CR: complete remission; PR: partial remission.
(DOCX)

S7 Table Baseline clinical characteristics of patients who received immunosuppression or supportive treatment only. DSC–doubling of serum creatinine; eGFR–estimated GFR according to the CKD-EPI formula; PLA_2_R1-ab–PLA_2_R1-antibody; CR–complete remission; PR–partial remission
(DOCX)

S8 Table Baseline clinical characteristics of patients who only received supportive treatment. Patients are grouped depending on whether PLA_2_R1-ab persisted in the circulation during follow-up. DSC–doubling of serum creatinine; eGFR–estimated GFR according to the CKD-EPI formula; PLA_2_R1-ab–PLA_2_R1-antibody; CR–complete remission; PR–partial remission
(DOCX)

S9 Table Clinical characteristics at baseline and the time of treatment start for patients who received immunosuppressive therapy. Patients are grouped depending on the first immunosuppressive treatment they received. In some cases immunosuppressive treatment was started at the time between two study visits, therefore data on proteinuria, serum creatinine and PLA_2_R1-ab levels were not available at the exact time when immunosuppression was started. These patients were not included in these analyses. Other immunosuppressants were only rarely used and therefore not included in these analyses. CYC: cyclophosphamide; CsA: cyclosporine A; RTX: rituximab; iv: intravenous; PLA_2_R1-ab: PLA_2_R1 antibody.
(DOCX)

S10 Table P-values for all differences of clinical characteristics at baseline and the time of treatment start shown in s9 Table. A: p-values for the comparisons between patients in the different treatment groups are presented. B: p-values for the comparisons between the clinical characteristics at baseline compared to the time of start of immunosuppression within the same treatment group are presented. CYC: cyclophosphamide; CsA: cyclosporine A; RTX: rituximab; iv: intravenous; PLA_2_R1-ab: PLA_2_R1 antibody.
(DOCX)

S11 Table Clinical baseline characteristics and outcomes of patients who needed a second line immunosuppressive treatment and those who did not. eGFR–estimated GFR according to the CKD-EPI formula; PLA_2_R1-ab–PLA_2_R1-antibody; CR–complete remission; PR–partial remission.
(DOCX)

S1 File Supplemental methods. (DOCX)
