# Supplementary material for: Role of phospholipase A2 receptor 1 antibody level at diagnosis for long-term renal outcome in membranous nephropathy
Source: PLoS One. 2019 Sep 9;14(9):e0221293. doi: 10.1371/journal.pone.0221293 (PMC6733455; doi:10.1371/journal.pone.0221293)
Supplement: S1 Fig — The cosine of the angle between the component loading vectors of the variables represents the correlation among the respective variables in the shown two-dimensional solution. An angle close to 0° indicates high positive correlation, an angle close to 180° indicates negative correlation, angles around 90° indicate no correlation. The length of a vector indicates the importance of the respective variable for the two-dimensional solution. eGFR: estimated GFR based on the CKD-EPI formula. PLA2R1-ab: PLA2R1 antibody. (DOCX) [file pone.0221293.s001.docx]

**
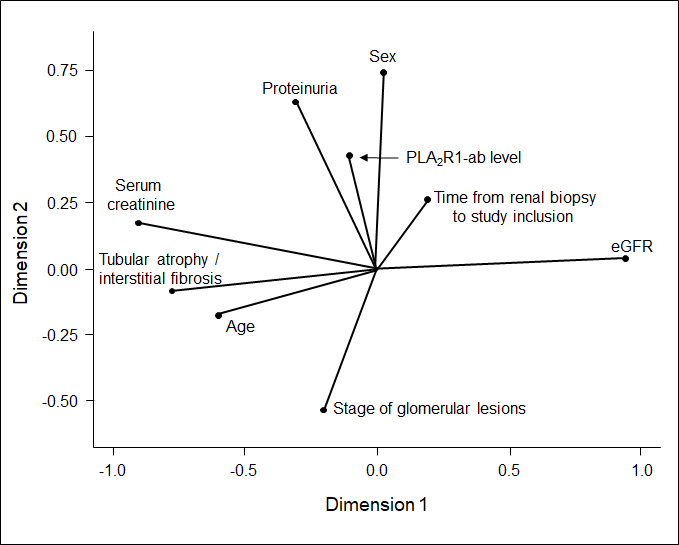
**

**S1 Fig. Component loading vectors of all clinical baseline variables.**

The cosine of the angle between the component loading vectors of the variables represents the correlation among the respective variables in the shown two-dimensional solution. An angle close to 0° indicates high positive correlation, an angle close to 180° indicates negative correlation, angles around 90° indicate no correlation. The length of a vector indicates the importance of the respective variable for the two-dimensional solution. eGFR: estimated GFR based on the CKD-EPI formula. PLA_2_R1-ab: PLA_2_R1 antibody.
