# Supplementary material for: Role of phospholipase A2 receptor 1 antibody level at diagnosis for long-term renal outcome in membranous nephropathy
Source: PLoS One. 2019 Sep 9;14(9):e0221293. doi: 10.1371/journal.pone.0221293 (PMC6733455; doi:10.1371/journal.pone.0221293)
Supplement: S2 Fig — A: PLA2R1-ab levels at baseline and use of immunosuppressive treatment were the only variables significantly associated with depletion of PLA2R1-ab during follow-up. The variable PLA2R1-ab level was transformed to its binary logarithm for this analysis. We adjusted the analysis for time-varying effects during follow-up and found a significant time-dependent change of the variable effect for both PLA2R1-ab levels and age. However, the effect of age for the endpoint was not significant. B: Use of immunosuppression was the only variable identified as a significant risk factor for relapse of PLA2R1-ab. 95%CI: 95% Confidence Interval; HR: hazard ratio; PLA2R1-ab: PLA2R1-antibody. (DOCX) [file pone.0221293.s002.docx]

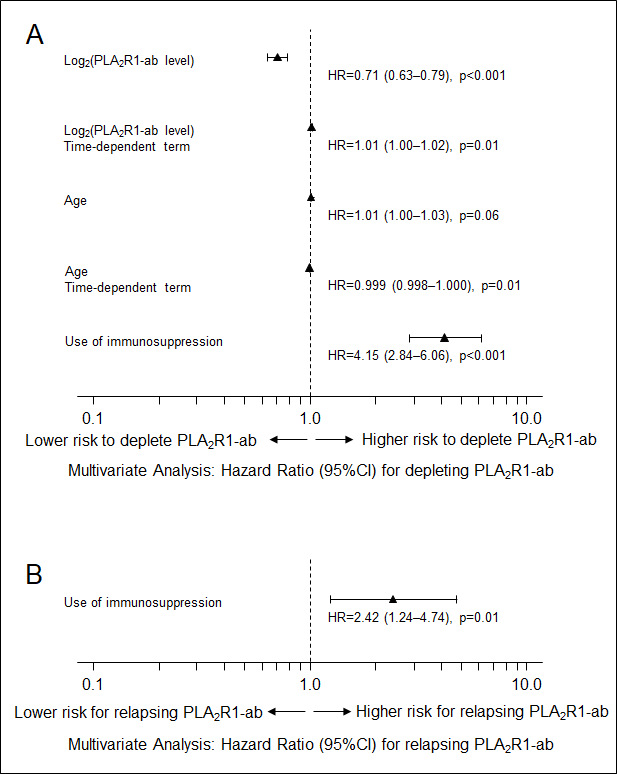


**S2 Fig. Multivariate Cox regression analysis for depletion and relapse of PLA_2_R1-ab.**

A: PLA_2_R1-ab levels at baseline and use of immunosuppressive treatment were the only variables significantly associated with depletion of PLA_2_R1-ab during follow-up. The variable PLA_2_R1-ab level was transformed to its binary logarithm for this analysis. We adjusted the analysis for time-varying effects during follow-up and found a significant time-dependent change of the variable effect for both PLA_2_R1-ab levels and age. However, the effect of age for the endpoint was not significant. B: Use of immunosuppression was the only variable identified as a significant risk factor for relapse of PLA_2_R1-ab. 95%CI: 95% Confidence Interval; HR: hazard ratio; PLA_2_R1-ab: PLA_2_R1-antibody.
