# Supplementary material for: Role of phospholipase A2 receptor 1 antibody level at diagnosis for long-term renal outcome in membranous nephropathy
Source: PLoS One. 2019 Sep 9;14(9):e0221293. doi: 10.1371/journal.pone.0221293 (PMC6733455; doi:10.1371/journal.pone.0221293)
Supplement: S3 Fig — A: PLA2R1-ab levels, proteinuria at baseline and depletion of PLA2R1-ab levels during follow-up were risk factors for remission of proteinuria. We adjusted the analysis for time-varying effects during follow-up and found a significant time-dependent change of the variable effect for both proteinuria and serum creatinine. However, the effect of serum creatinine for the study end point was not significant. B: PLA2R1-ab levels, serum creatinine at baseline, relapse of PLA2R1-ab levels and partial remission of proteinuria (compared to complete remission) were identified as significant risk factors for relapse of proteinuria. The variables PLA2R1-ab level, proteinuria, and serum creatinine were transformed to their binary logarithm prior to using them in the Cox regression analyses. 95% CI: 95% confidence interval; HR: hazard ratio; PLA2R1-ab: PLA2R1-antibody. (DOCX) [file pone.0221293.s003.docx]

**
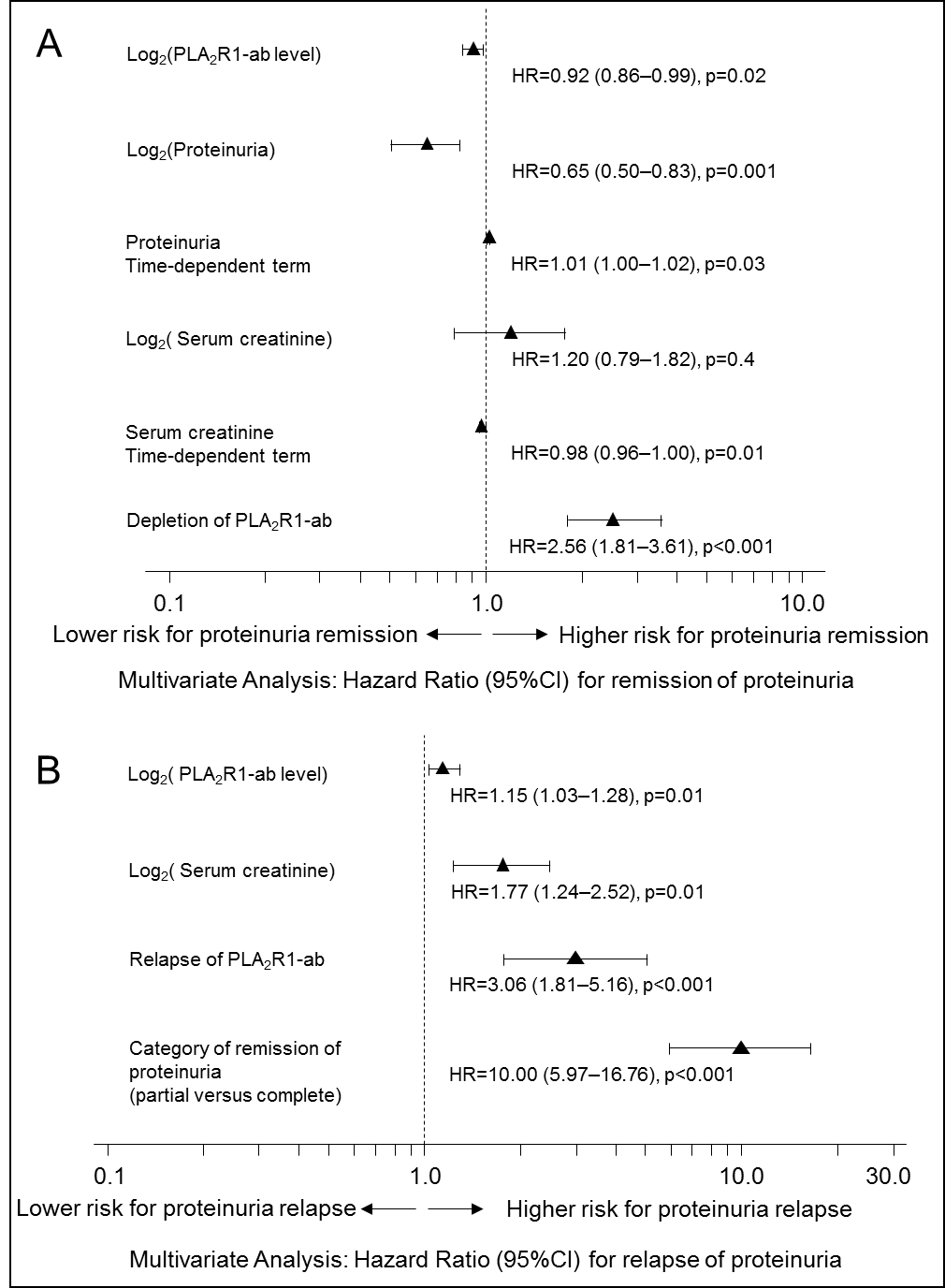
**

**S3 Fig. Multivariate Cox regression analysis for remission and relapse of proteinuria**

A: PLA_2_R1-ab levels, proteinuria at baseline and depletion of PLA_2_R1-ab levels during follow-up were risk factors for remission of proteinuria. We adjusted the analysis for time-varying effects during follow-up and found a significant time-dependent change of the variable effect for both proteinuria and serum creatinine. However, the effect of serum creatinine for the study end point was not significant. B: PLA_2_R1-ab levels, serum creatinine at baseline, relapse of PLA_2_R1-ab levels and partial remission of proteinuria (compared to complete remission) were identified as significant risk factors for relapse of proteinuria. The variables PLA_2_R1-ab level, proteinuria, and serum creatinine were transformed to their binary logarithm prior to using them in the Cox regression analyses. 95% CI: 95% confidence interval; HR: hazard ratio; PLA_2_R1-ab: PLA_2_R1-antibody.
