# Supplementary material for: Role of phospholipase A2 receptor 1 antibody level at diagnosis for long-term renal outcome in membranous nephropathy
Source: PLoS One. 2019 Sep 9;14(9):e0221293. doi: 10.1371/journal.pone.0221293 (PMC6733455; doi:10.1371/journal.pone.0221293)
Supplement: S1 Table — eGFR–estimated GFR according to the CKD-EPI formula; PLA2R1-ab–PLA2R1-antibody. (DOCX) [file pone.0221293.s004.docx]

**S1 Table. Clinical baseline characteristics and outcomes of patients with eGFR below or higher than 60 mL/min/1.73 m^2^ at baseline.**

|  | | **eGFR < 60 mL/min/1.73 m^2^** | **eGFR > 60 mL/min/1.73 m^2^** | **P-value** |
| --- | --- | --- | --- | --- |
| **Number of Patients** | | 63 | 180 | na |
| **Age - years**  **(median, 1^st^ - 3^rd^ quartile)** | | 68.0,  60.5 – 74.5 | 52.0,  41.0 – 60.0 | <0.001 |
| **Male sex (%)** | | 38 (60%) | 133 (74%) | 0.01 |
| **Proteinuria - g/24h**  **(median, 1^st^ - 3^rd^ quartile)** | | 8.4,  5.9 – 11.1 | 6.7,  4.3 – 10.0 | 0.02 |
| **Serum creatinine - mg/dl**  **(median, 1^st^ - 3^rd^ quartile)** | | 1.7,  1.4 – 2.1 | 0.9,  0.8 – 1.1 | <0.001 |
| **eGFR, CKD-EPI - mL/min/1.73 m^2^ (median, 1^st^ - 3^rd^ quartile)** | | 38.2,  29.2 – 45.4 | 92.5,  78.7 – 105.8 | <0.001 |
| **PLA_2_R1-ab level, U/ml**  **(median, 1^st^ - 3^rd^ quartile)** | | 150.9,  64.3 – 394.3 | 118.7,  56.6 – 250.5 | 0.3 |
| **Time between renal biopsy and study inclusion - months (median, 1^st^ - 3^rd^ quartile)** | | 0.3,  0.0 – 1.0 | 0.8,  0.3 – 1.0 | 0.04 |
| **% of tubulointerstitial space with tubular atrophy and interstitial fibrosis** | | 20,  10 – 50 | 5,  0 – 10 | <0.001 |
| **Immunosuppressive treatment during follow-up (%)** | | 55 (87%) | 134 (74%) | 0.04 |
| **PLA_2_R1-ab persistent throughout the follow-up (%)** | | 14 (22%) | 35 (19%) | 0.7 |
| **Relapse of PLA_2_R1-ab during follow-up (%)** | | 16 (25%) | 56 (31%) | 0.4 |
| **Remission of proteinuria** | **CR (%)** | 31 (49%) | 104 (58%) | 0.2 |
|  | **PR (%)** | 18 (29%) | 63 (35%) | 0.4 |
| **Doubling of serum creatinine (%)** | | 19 (30%) | 17 (9%) | <0.001 |

eGFR – estimated GFR according to the CKD-EPI formula; PLA_2_R1-ab – PLA_2_R1-antibody.
