# Supplementary material for: Role of phospholipase A2 receptor 1 antibody level at diagnosis for long-term renal outcome in membranous nephropathy
Source: PLoS One. 2019 Sep 9;14(9):e0221293. doi: 10.1371/journal.pone.0221293 (PMC6733455; doi:10.1371/journal.pone.0221293)
Supplement: S2 Table — In the analyses of independent variables measured at baseline we adjusted the analysis for potential time-varying effects during follow-up, while for independent variables representing events measured during follow-up we adjusted the effect of the variable for the time when its event occurred. This table presents results of each variable both with and without adjusting for these time-dependent effects. Un-adjusted analyses consider only a main effect term for each variable. Analyses of baseline variables which were adjusted for time-varying effects consider a main effect term (reflecting the initial effect of the variable) and a time-dependent term (reflecting the change of the variable effect during time). Analyses adjusted for time-varying effects of event variables measured during follow-up consider only a time-dependent term (reflecting the effect of the variable from the time when its event occurs). 95% Conf. Interval: 95% Confidence Interval; PLA2R1-ab: PLA2R1-antibody; Time-dep.: time-dependent; CR: complete remission; PR: partial remission. (DOCX) [file pone.0221293.s005.docx]

**S2 Table. Univariate Cox regression analysis to identify clinical parameters predictive for the study endpoint.**

| **Variable** | **Analysis is adjusted for time-dependent effects** | **Term** | **Hazard Ratio** | **95% Conf. Interval** | | **P-value** |
| --- | --- | --- | --- | --- | --- | --- |
|  |  |  |  | **Lower** | **Upper** |  |
| **Log_2_(PLA_2_R1-ab level)** | No | Main effect | 1.45 | 1.19 | 1.77 | <0.001 |
|  | Yes | Main effect | 1.75 | 1.23 | 2.48 | 0.01 |
|  | Yes | Time-dep. | 0.99 | 0.98 | 1.00 | 0.2 |
| **Log_2_(Proteinuria)** | No | Main effect | 0.93 | 0.65 | 1.32 | 0.7 |
|  | Yes | Main effect | 0.76 | 0.42 | 1.38 | 0.4 |
|  | Yes | Time-dep. | 1.01 | 0.99 | 1.03 | 0.4 |
| **Log_2_(Serum creatinine)** | No | Main effect | 2.81 | 1.78 | 4.43 | <0.001 |
|  | Yes | Main effect | 1.89 | 0.84 | 4.29 | 0.1 |
|  | Yes | Time-dep. | 1.02 | 0.99 | 1.04 | 0.2 |
| **Age** | No | Main effect | 1.02 | 1.00 | 1.04 | 0.08 |
|  | Yes | Main effect | 1.00 | 0.97 | 1.04 | 0.8 |
|  | Yes | Time-dep. | 1.00 | 1.00 | 1.00 | 0.4 |
| **Sex** | No | Main effect | 1.01 | 0.48 | 2.09 | 0.9 |
|  | Yes | Main effect | 0.70 | 0.20 | 2.44 | 0.6 |
|  | Yes | Time-dep. | 1.02 | 0.97 | 1.06 | 0.5 |
| **Log_2_(% of tubule-interstitial space with tubular atrophy and interstitial fibrosis**) | No | Main effect | 1.51 | 1.24 | 1.85 | <0.001 |
|  | Yes | Main effect | 1.85 | 1.27 | 2.70 | 0.001 |
|  | Yes | Time-dep. | 0.99 | 0.98 | 1.00 | 0.2 |
| **Log_2_(Time between renal biopsy and study enrolment)** | No | Main effect | 0.98 | 0.82 | 1.17 | 0.8 |
|  | Yes | Main effect | 0.92 | 0.67 | 1.26 | 0.6 |
|  | Yes | Time-dep. | 1.00 | 0.99 | 1.01 | 0.6 |
| **Depletion of PLA_2_R1-ab** | No | Main effect | 0.22 | 0.11 | 0.44 | <0.001 |
|  | Yes | Time-dep. | 0.40 | 0.19 | 0.85 | 0.02 |
| **Relapse of PLA_2_R1-ab** | No | Main effect | 3.01 | 1.41 | 6.41 | 0.01 |
|  | Yes | Time-dep. | 4.68 | 2.26 | 9.67 | <0.001 |
| **Remission of proteinuria** | No | Main effect | 0.17 | 0.08 | 0.35 | <0.001 |
|  | Yes | Time-dep. | 0.81 | 0.36 | 1.79 | 0.6 |
| **Category of remission (PR compared to CR)** | No | Main effect | 3.74 | 1.85 | 7.57 | <0.001 |
|  | Yes | Time-dep. | 2.16 | 1.07 | 4.37 | 0.03 |
| **Relapse of proteinuria** | No | Main effect | 6.23 | 2.83 | 13.68 | <0.001 |
|  | Yes | Time-dep. | 6.45 | 3.30 | 12.64 | <0.001 |
| **Use of immunosuppressive treatment** | No | Main effect | 1.68 | 0.65 | 4.33 | 0.3 |
|  | Yes | Time-dep. | 2.07 | 0,84 | 5.08 | 0.1 |

In the analyses of independent variables measured at baseline we adjusted the analysis for potential time-varying effects during follow-up, while for independent variables representing events measured during follow-up we adjusted the effect of the variable for the time when its event occurred. This table presents results of each variable both with and without adjusting for these time-dependent effects. Un-adjusted analyses consider only a main effect term for each variable. Analyses of baseline variables which were adjusted for time-varying effects consider a main effect term (reflecting the initial effect of the variable) and a time-dependent term (reflecting the change of the variable effect during time). Analyses adjusted for time-varying effects of event variables measured during follow-up consider only a time-dependent term (reflecting the effect of the variable from the time when its event occurs). 95% Conf. Interval: 95% Confidence Interval; PLA_2_R1-ab: PLA_2_R1-antibody; Time-dep.: time-dependent; CR: complete remission; PR: partial remission.
