# Supplementary material for: Role of phospholipase A2 receptor 1 antibody level at diagnosis for long-term renal outcome in membranous nephropathy
Source: PLoS One. 2019 Sep 9;14(9):e0221293. doi: 10.1371/journal.pone.0221293 (PMC6733455; doi:10.1371/journal.pone.0221293)
Supplement: S3 Table — In the analyses of independent variables measured at baseline we adjusted the analysis for potential time-varying effects during follow-up. In this table we present results of each variable both with, and without adjusting for these time-dependent effects. Unadjusted analyses consider only a main effect term for each variable. Analyses of baseline variables which were adjusted for time-varying effects consider a main effect term (reflecting the initial effect of the variable) and a time-dependent term (reflecting the change of the variable effect during time). In the analysis of “use of immunosuppressive treatment” when adjusting for time-varying effects we consider only a time-dependent term (reflecting the effect of the variable from the time when the event occurs–immunosuppression is started). 95% Conf. Interval: 95% Confidence Interval; PLA2R1-ab: PLA2R1-antibody; Time-dep.: time-dependent. (DOCX) [file pone.0221293.s006.docx]

**S3 Table. Univariate Cox regression analysis for depletion of PLA_2_R1-ab.**

| **Variable** | **Analysis is adjusted for time-dependent effects** | **Term** | **Hazard Ratio** | **95% Conf. Interval** | | **P-value** |
| --- | --- | --- | --- | --- | --- | --- |
|  |  |  |  | **Lower** | **Upper** |  |
| **Log_2_(PLA_2_R1-ab level)** | No | Main effect | 0.82 | 0.76 | 0.88 | <0.001 |
|  | Yes | Main effect | 0.74 | 0.66 | 0.82 | <0.001 |
|  | Yes | Time-dep. | 1.01 | 1.00 | 1.02 | 0.01 |
| **Log_2_(Proteinuria)** | No | Main effect | 1.19 | 1.00 | 1.41 | 0.04 |
|  | Yes | Main effect | 1.11 | 0.86 | 1.43 | 0.4 |
|  | Yes | Time-dep. | 1.01 | 0.99 | 1.03 | 0.5 |
| **Log_2_(Serum creatinine)** | No | Main effect | 1.04 | 0.82 | 1.31 | 0.7 |
|  | Yes | Main effect | 1.40 | 1.00 | 1.95 | 0.05 |
|  | Yes | Time-dep. | 0.98 | 0.96 | 1.00 | 0.02 |
| **Age** | No | Main effect | 1.00 | 0.99 | 1.01 | 0.7 |
|  | Yes | Main effect | 1.01 | 1.00 | 1.02 | 0.1 |
|  | Yes | Time-dep. | 1.00 | 1.00 | 1.00 | 0.02 |
| **Sex** | No | Main effect | 1.16 | 0.85 | 1.58 | 0.4 |
|  | Yes | Main effect | 1.09 | 0.69 | 1.72 | 0.7 |
|  | Yes | Time-dep. | 1.01 | 0.98 | 1.04 | 0.7 |
| **Log_2_(Time between renal biopsy and study enrolment)** | No | Main effect | 1.01 | 0.94 | 1.09 | 0.7 |
|  | Yes | Main effect | 0.99 | 0.88 | 1.10 | 0.8 |
|  | Yes | Time-dep. | 1.00 | 1.00 | 1.01 | 0.5 |
| **Use of immunosuppressive treatment** | No | Main effect | 1.16 | 0.82 | 1.64 | 0.4 |
|  | Yes | Time-dep. | 3.55 | 2.44 | 5.17 | <0.001 |

In the analyses of independent variables measured at baseline we adjusted the analysis for potential time-varying effects during follow-up. In this table we present results of each variable both with, and without adjusting for these time-dependent effects. Unadjusted analyses consider only a main effect term for each variable. Analyses of baseline variables which were adjusted for time-varying effects consider a main effect term (reflecting the initial effect of the variable) and a time-dependent term (reflecting the change of the variable effect during time). In the analysis of “use of immunosuppressive treatment” when adjusting for time-varying effects we consider only a time-dependent term (reflecting the effect of the variable from the time when the event occurs – immunosuppression is started). 95% Conf. Interval: 95% Confidence Interval; PLA_2_R1-ab: PLA_2_R1-antibody; Time-dep.: time-dependent.
