# Supplementary material for: Role of phospholipase A2 receptor 1 antibody level at diagnosis for long-term renal outcome in membranous nephropathy
Source: PLoS One. 2019 Sep 9;14(9):e0221293. doi: 10.1371/journal.pone.0221293 (PMC6733455; doi:10.1371/journal.pone.0221293)
Supplement: S5 Table — In the analyses of independent variables measured at baseline we adjusted the analysis for potential time-varying effects during follow-up. In this table we present results of each variable both with, and without adjusting for these time-dependent effects. Unadjusted analyses consider only a main effect term for each variable. Analyses of baseline variables which were adjusted for time-varying effects consider a main effect term (reflecting the initial effect of the variable) and a time-dependent term (reflecting the change of the variable effect during time). Analyses adjusted for time-varying effects of event variables measured during follow-up consider only a time-dependent term (reflecting the effect of the variable from the time when its event occurs). 95% Conf. Interval: 95% Confidence Interval; PLA2R1-ab: PLA2R1-antibody; Time-dep.: time-dependent. (DOCX) [file pone.0221293.s008.docx]

**S5 Table. Univariate Cox regression analysis for remission of proteinuria.**

| **Variable** | **Analysis is adjusted for time-dependent effects** | **Term** | **Hazard Ratio** | **95% Conf. Interval** | | **P-value** |
| --- | --- | --- | --- | --- | --- | --- |
|  |  |  |  | **Lower** | **Upper** |  |
| **Log_2_(PLA_2_R1-ab level)** | No | Main effect | 0.87 | 0.81 | 0.93 | <0.001 |
|  | Yes | Main effect | 0.85 | 0.77 | 0.94 | 0.01 |
|  | Yes | Time-dep. | 1.00 | 1.00 | 1.01 | 0.6 |
| **Log_2_(Proteinuria)** | No | Main effect | 0.79 | 0.68 | 0.90 | 0.001 |
|  | Yes | Main effect | 0.63 | 0.50 | 0.80 | <0.001 |
|  | Yes | Time-dep. | 1.01 | 1.00 | 1.02 | 0.03 |
| **Log_2_(Serum creatinine)** | No | Main effect | 0.69 | 0.54 | 0.87 | 0.01 |
|  | Yes | Main effect | 1.01 | 0.69 | 1.47 | 0.9 |
|  | Yes | Time-dep. | 0.98 | 0.97 | 1.00 | 0.02 |
| **Age** | No | Main effect | 0.99 | 0.98 | 1.00 | 0.06 |
|  | Yes | Main effect | 1.00 | 0.98 | 1.01 | 0.5 |
|  | Yes | Time-dep. | 1.00 | 1.00 | 1.00 | 0.5 |
| **Sex** | No | Main effect | 0.96 | 0.71 | 1.29 | 0.8 |
|  | Yes | Main effect | 0.84 | 0.53 | 1.33 | 0.5 |
|  | Yes | Time-dep. | 1.01 | 0.99 | 12.03 | 0.5 |
| **Log_2_(% of tubulointerstitial space with tubular atrophy and interstitial fibrosis**) | No | Main effect | 0.94 | 0.88 | 1.01 | 0.08 |
|  | Yes | Main effect | 1.00 | 0.90 | 1.12 | 0.9 |
|  | Yes | Time-dep. | 1.00 | 0.99 | 1.00 | 0.2 |
| **Log_2_(Time between renal biopsy and study enrolment)** | No | Main effect | 1.03 | 0.96 | 1.11 | 0.4 |
|  | Yes | Main effect | 0.96 | 0.85 | 1.08 | 0.5 |
|  | Yes | Time-dep. | 1.00 | 1.00 | 1.01 | 0.1 |
| **Depletion of PLA_2_R1-ab** | No | Main effect | 1.19 | 0.82 | 1.74 | 0.4 |
|  | Yes | Time-dep. | 2.67 | 1.91 | 3.75 | <0.001 |
| **Relapse of PLA_2_R1-ab** | No | Main effect | 0.89 | 0.68 | 1.17 | 0.4 |
|  | Yes | Time-dep. | 0.89 | 0.65 | 1.22 | 0.5 |
| **Use of immunosuppressive treatment** | No | Main effect | 0.91 | 0.66 | 1.26 | 0.6 |
|  | Yes | Time-dep. | 1.31 | 0.94 | 1.83 | 0.1 |

In the analyses of independent variables measured at baseline we adjusted the analysis for potential time-varying effects during follow-up. In this table we present results of each variable both with, and without adjusting for these time-dependent effects. Unadjusted analyses consider only a main effect term for each variable. Analyses of baseline variables which were adjusted for time-varying effects consider a main effect term (reflecting the initial effect of the variable) and a time-dependent term (reflecting the change of the variable effect during time). Analyses adjusted for time-varying effects of event variables measured during follow-up consider only a time-dependent term (reflecting the effect of the variable from the time when its event occurs). 95% Conf. Interval: 95% Confidence Interval; PLA_2_R1-ab: PLA_2_R1-antibody; Time-dep.: time-dependent.
