# Supplementary material for: Role of phospholipase A2 receptor 1 antibody level at diagnosis for long-term renal outcome in membranous nephropathy
Source: PLoS One. 2019 Sep 9;14(9):e0221293. doi: 10.1371/journal.pone.0221293 (PMC6733455; doi:10.1371/journal.pone.0221293)
Supplement: S7 Table — DSC–doubling of serum creatinine; eGFR–estimated GFR according to the CKD-EPI formula; PLA2R1-ab–PLA2R1-antibody; CR–complete remission; PR–partial remission (DOCX) [file pone.0221293.s010.docx]

**S7 Table. Baseline clinical characteristics of patients who received immunosuppression or supportive treatment only.**

|  | | **Immunosuppressive treatment** | **Only supportive treatment** | **P-value** |
| --- | --- | --- | --- | --- |
| **Number of Patients** | | 189 | 54 | Na |
| **Age - years**  **(median, 1^st^ - 3^rd^ quartile)** | | 55.0,  43.0 – 66.0 | 57.5,  41.5 – 64.0 | 0.9 |
| **Male sex (%)** | | 140 (74%) | 31 (57%) | 0.03 |
| **Proteinuria - g/24h**  **(median, 1^st^ - 3^rd^ quartile)** | | 8.0,  5.1 – 10.9 | 5.1,  3.0 – 7.1 | <0.001 |
| **Serum creatinine - mg/dl (median, 1^st^ - 3^rd^ quartile)** | | 1.0,  0.9 – 1.4 | 0.9,  0.7 – 1.1 | 0.01 |
| **eGFR, CKD-EPI - mL/min/1.73 m^2^**  **(median, 1^st^ - 3^rd^ quartile)** | | 81.2,  53.2 – 98.2 | 90.1,  72.3 – 104.5 | 0.03 |
| **PLA_2_R1-ab level, U/ml**  **(median, 1^st^ - 3^rd^ quartile)** | | 155.3,  77.3 – 321.2 | 50.1,  11.0 – 119.7 | <0.001 |
| **Time between renal biopsy and study inclusion - months (median, 1^st^ - 3^rd^ quartile)** | | 0.5,  0.0 – 1.0 | 0.5,  0.3 – 1.0 | 0.6 |
| **% of tubulointerstitial space with tubular atrophy and interstitial fibrosis** | | 5.0,  0.0 – 20.0 | 7.5,  5.0 – 13.8 | 0.9 |
| **PLA_2_R1-ab persistent throughout the follow-up (%)** | | 36 (19%) | 13 (24%) | 0.4 |
| **Relapse of PLA_2_R1-ab during follow-up (%)** | | 62 (33%) | 10 (19%) | 0.04 |
| **Remission of proteinuria** | **CR (%)** | 106 (56%) | 29 (54%) | 0.8 |
|  | **PR (%)** | 62 (33%) | 11 (27%) | 0.7 |
| **DSC (%)** | | 31 (16%) | 5 (9%) | 0.3 |

DSC – doubling of serum creatinine; eGFR – estimated GFR according to the CKD-EPI formula; PLA_2_R1-ab – PLA_2_R1-antibody; CR – complete remission; PR – partial remission
