# Supplementary material for: Role of phospholipase A2 receptor 1 antibody level at diagnosis for long-term renal outcome in membranous nephropathy
Source: PLoS One. 2019 Sep 9;14(9):e0221293. doi: 10.1371/journal.pone.0221293 (PMC6733455; doi:10.1371/journal.pone.0221293)
Supplement: S8 Table — Patients are grouped depending on whether PLA2R1-ab persisted in the circulation during follow-up. DSC–doubling of serum creatinine; eGFR–estimated GFR according to the CKD-EPI formula; PLA2R1-ab–PLA2R1-antibody; CR–complete remission; PR–partial remission (DOCX) [file pone.0221293.s011.docx]

**S8 Table. Baseline clinical characteristics of patients who only received supportive treatment.**

|  | | **Spontaneous reduction of PLA_2_R1-ab** | **Persistent PLA_2_R1-ab during follow-up** | **P-value** |
| --- | --- | --- | --- | --- |
| **Number of Patients** | | 41 | 13 | na |
| **Age - years**  **(median, 1^st^ - 3^rd^ quartile)** | | 54.0,  37.0 – 62.0 | 62.0,  57.0 – 66.0 | 0.1 |
| **Male sex (%)** | | 21 (51%) | 10 (77%) | 0.1 |
| **Proteinuria - g/24h**  **(median, 1^st^ - 3^rd^ quartile)** | | 4.8,  2.6 – 7.0 | 6.0,  4.3 – 7.8 | 0.2 |
| **Serum creatinine - mg/dl (median, 1^st^ - 3^rd^ quartile)** | | 0.9,  0.7 – 1.1 | 0.8,  0.8 – 1.9 | 0.5 |
| **eGFR, CKD-EPI - mL/min/1.73 m^2^**  **(median, 1^st^ - 3^rd^ quartile)** | | 87.7,  73.2 – 105.7 | 93.5,  42.2 – 98.8 | 0.5 |
| **PLA_2_R1-ab level, U/ml**  **(median, 1^st^ - 3^rd^ quartile)** | | 21.2,  5.8 – 79.5 | 89.4,  52.6 – 158.7 | 0.05 |
| **Time between renal biopsy and study inclusion - months (median, 1^st^ - 3^rd^ quartile)** | | 0.5,  0.3 – 1.0 | 0.5,  0.0 – 1.0 | 0.2 |
| **Relapse of PLA_2_R1-ab during follow-up (%)** | | 10 (24%) | na | na |
| **Remission of proteinuria** | **CR (%)** | 29 (71%) | 0 (0%) | <0.001 |
|  | **PR (%)** | 11 (27%) | 8 (62%) | 0.04 |
| **DSC (%)** | | 2 (5%) | 3 (23%) | 0.08 |

Patients are grouped depending on whether PLA_2_R1-ab persisted in the circulation during follow-up. DSC – doubling of serum creatinine; eGFR – estimated GFR according to the CKD-EPI formula; PLA_2_R1-ab – PLA_2_R1-antibody; CR – complete remission; PR – partial remission.
