# Supplementary material for: Role of phospholipase A2 receptor 1 antibody level at diagnosis for long-term renal outcome in membranous nephropathy
Source: PLoS One. 2019 Sep 9;14(9):e0221293. doi: 10.1371/journal.pone.0221293 (PMC6733455; doi:10.1371/journal.pone.0221293)
Supplement: S9 Table — Patients are grouped depending on the first immunosuppressive treatment they received. In some cases immunosuppressive treatment was started at the time between two study visits, therefore data on proteinuria, serum creatinine and PLA2R1-ab levels were not available at the exact time when immunosuppression was started. These patients were not included in these analyses. Other immunosuppressants were only rarely used and therefore not included in these analyses. CYC: cyclophosphamide; CsA: cyclosporine A; RTX: rituximab; iv: intravenous; PLA2R1-ab: PLA2R1 antibody. (DOCX) [file pone.0221293.s012.docx]

|  | | **CYC (oral)** | **CYC (iv)** | **CsA** | **RTX** |
| --- | --- | --- | --- | --- | --- |
| **Number of Patients** | | 39 | 35 | 81 | 19 |
| **Age - years (median, 1^st^ – 3^rd^ quartile)** | | 54.0, 45.5 – 63.5 | 61.0, 51.5 – 73.5 | 53.0, 38.0 – 65.0 | 59.0, 44.5 – 66.5 |
| **Male Gender (%)** | | 33 (84.6%) | 21 (60.0%) | 65 (80.2%) | 11 (57.9%) |
| **Time from study inclusion to start of treatment - months (median, 1^st^ – 3^rd^ quartile)** | | 3.0, 0.0 – 6.0 | 0.0, 0.0 – 3.0 | 3.0, 0.0 – 6.0 | 0.0, 0.0 – 4.5 |
| **Change of immunosuppressive treatment during follow-up (%)** | | 18 (46.2%) | 20 (57.1%) | 42 (51.9%) | 8 (42.1%) |
| **Proteinuria - g/24h**  **(median, 1^st^ – 3^rd^ quartile)** | **At Baseline** | 10.7, 8.9 – 11.3 | 8.7, 4.8 – 11.0 | 7.9, 6.0 – 11.2 | 6.4, 3.9 – 9.5 |
|  | **At start of immunosuppression** | 10.6, 6.7 – 15.3 | 9.0, 5.8 – 13.0 | 9.0, 6.3 – 13.0 | 6.8, 4.0 – 10.6 |
| **Serum creatinine - mg/dl (median, 1^st^ – 3^rd^ quartile)** | **At Baseline** | 1.1, 0.9 – 1.6 | 1.1, 0.8 – 1.6 | 1.1, 0.9 – 1.2 | 1.0, 0.9 – 1.4 |
|  | **At start of immunosuppression** | 1.3, 1.0 – 1.8 | 1.3, 1.0 – 1.7 | 1.1, 0.9 – 1.5 | 1.1, 1.0 – 1.7 |
| **PLA_2_R1-ab level, U/ml (median, 1^st^ – 3^rd^ quartile)** | **At Baseline** | 175, 92 – 195 | 134, 86 – 312 | 130, 67 – 335 | 104, 59 – 164 |
|  | **At start of immunosuppression** | 135, 56 – 280 | 107, 42 – 321 | 106, 50 – 259 | 103, 64 – 234 |

**S9 Table. Clinical characteristics at baseline and the time of treatment start for patients who received immunosuppressive therapy.**

Patients are grouped depending on the first immunosuppressive treatment they received. In some cases immunosuppressive treatment was started at the time between two study visits, therefore data on proteinuria, serum creatinine and PLA_2_R1-ab levels were not available at the exact time when immunosuppression was started. These patients were not included in these analyses. Other immunosuppressants were only rarely used and therefore not included in these analyses. CYC: cyclophosphamide; CsA: cyclosporine A; RTX: rituximab; iv: intravenous; PLA_2_R1-ab: PLA_2_R1 antibody.
