# Supplementary material for: Role of phospholipase A2 receptor 1 antibody level at diagnosis for long-term renal outcome in membranous nephropathy
Source: PLoS One. 2019 Sep 9;14(9):e0221293. doi: 10.1371/journal.pone.0221293 (PMC6733455; doi:10.1371/journal.pone.0221293)
Supplement: S10 Table — A: p-values for the comparisons between patients in the different treatment groups are presented. B: p-values for the comparisons between the clinical characteristics at baseline compared to the time of start of immunosuppression within the same treatment group are presented. CYC: cyclophosphamide; CsA: cyclosporine A; RTX: rituximab; iv: intravenous; PLA2R1-ab: PLA2R1 antibody. (DOCX) [file pone.0221293.s013.docx]

**S10 Table. P-values for all differences of clinical characteristics at baseline and the time of treatment start shown in supplemental Table 9.**

|  | | **CYC (oral) vs. CYC (iv)** | **CYC (oral) vs. CsA** | **CYC (oral) vs. RTX** | **CYC (iv)**  **vs. CsA** | **CYC (iv)**  **vs. RTX** | **CsA**  **vs. RTX** |
| --- | --- | --- | --- | --- | --- | --- | --- |
| **Age** | | 0.04 | 0.3 | 0.6 | 0.01 | 0.2 | 0.3 |
| **Gender** | | 0.02 | 0.6 | 0.05 | 0.04 | 0.9 | 0.07 |
| **Time from study inclusion to start of treatment** | | 0.1 | 0.4 | 0.2 | 0.4 | 0.9 | 0.5 |
| **Change of immunosuppressive treatment during follow-up** | | 0.4 | 0.7 | 0.9 | 0.7 | 0.4 | 0.6 |
| **Proteinuria** | **At baseline** | 0.1 | 0.03 | 0.01 | 0.9 | 0.3 | 0.2 |
|  | **At start of immunosuppression** | 0.6 | 0.7 | 0.2 | 0.8 | 0.3 | 0.1 |
| **Serum creatinine** | **At baseline** | 0.9 | 0.2 | 0.5 | 0.3 | 0.8 | 0.7 |
|  | **At start of immunosuppression** | 0.5 | 0.04 | 0.3 | 0.3 | 0.6 | 0.7 |
| **PLA_2_R1-ab** | **At baseline** | 0.9 | 0.7 | 0.2 | 0.9 | 0.4 | 0.3 |
|  | **At start of immunosuppression** | 0.8 | 0.8 | 0.9 | 0.7 | 0.8 | 0.9 |

**A**

**B**

|  | **CYC (oral)** | **CYC (iv)** | **CsA** | **RTX** |
| --- | --- | --- | --- | --- |
| **Proteinuria at baseline versus at start of immunosuppression** | 0.9 | 0.7 | 0.1 | 0.7 |
| **Serum creatinine at baseline versus at start of immunosuppression** | 0.1 | 0.4 | 0.09 | 0.4 |
| **PLA_2_R1-ab at baseline versus at start of immunosuppression** | 0.7 | 0.5 | 0.4 | 0.9 |

A: p-values for the comparisons between patients in the different treatment groups are presented. B: p-values for the comparisons between the clinical characteristics at baseline compared to the time of start of immunosuppression within the same treatment group are presented. CYC: cyclophosphamide; CsA: cyclosporine A; RTX: rituximab; iv: intravenous; PLA_2_R1-ab: PLA_2_R1 antibody.
