# Supplementary material for: Role of phospholipase A2 receptor 1 antibody level at diagnosis for long-term renal outcome in membranous nephropathy
Source: PLoS One. 2019 Sep 9;14(9):e0221293. doi: 10.1371/journal.pone.0221293 (PMC6733455; doi:10.1371/journal.pone.0221293)
Supplement: S11 Table — eGFR–estimated GFR according to the CKD-EPI formula; PLA2R1-ab–PLA2R1-antibody; CR–complete remission; PR–partial remission. (DOCX) [file pone.0221293.s014.docx]

|  | | **No second line immunosuppressive therapy** | **Second line immunosuppressive therapy** | **P-value** |
| --- | --- | --- | --- | --- |
| **Number of Patients** | | 93 | 96 | na |
| **Age - years**  **(median, 1^st^ - 3^rd^ quartile)** | | 55.0,  42.0 – 66.3 | 54.0,  43.0 – 64.0 | 0.7 |
| **Male sex (%)** | | 67 (70%) | 73 (78%) | 0.6 |
| **Proteinuria - g/24h**  **(median, 1^st^ - 3^rd^ quartile)** | | 7.3,  5.0 – 10.7 | 8.5,  5.8 – 11.2 | 0.3 |
| **Serum creatinine - mg/dl**  **(median, 1^st^ - 3^rd^ quartile)** | | 1.0,  0.8 – 1.4 | 1.1,  0.9 – 1.3 | 0.5 |
| **eGFR, CKD-EPI - mL/min/1.73 m^2^ (median, 1^st^ - 3^rd^ quartile)** | | 79.0,  45.3 – 102.9 | 81.8,  56.7 – 95.1 | 0.9 |
| **PLA_2_R1-ab level, U/ml**  **(median, 1^st^ - 3^rd^ quartile)** | | 164.2,  79.8 – 309.2 | 147.1,  74.3 – 365.1 | 0.7 |
| **Time between renal biopsy and study inclusion - months (median, 1^st^ - 3^rd^ quartile)** | | 0.8,  0.0 – 1.0 | 0.5,  0.0 – 1.0 | 0.5 |
| **% of tubulointerstitial space with tubular atrophy and interstitial fibrosis** | | 5,  5 – 20 | 5,  0 – 20 | 0.3 |
| **PLA_2_R1-ab persistent throughout the follow-up (%)** | | 19 (20%) | 17 (18%) | 0.7 |
| **Relapse of PLA_2_R1-ab during follow-up (%)** | | 36 (39%) | 26 (27%) | 0.1 |
| **Remission of proteinuria** | **CR (%)** | 49 (53%) | 57 (59%) | 0.4 |
|  | **PR (%)** | 33 (35%) | 29 (30%) | 0.5 |
| **Doubling of serum creatinine (%)** | | 17 (18%) | 14 (15%) | 0.6 |

**S11 Table. Clinical baseline characteristics and outcomes of patients who needed a second line immunosuppressive treatment and those who did not.**

eGFR – estimated GFR according to the CKD-EPI formula; PLA_2_R1-ab – PLA_2_R1-antibody; CR – complete remission; PR – partial remission.
