# Supplementary material for: Role of phospholipase A2 receptor 1 antibody level at diagnosis for long-term renal outcome in membranous nephropathy
Source: PLoS One. 2019 Sep 9;14(9):e0221293. doi: 10.1371/journal.pone.0221293 (PMC6733455; doi:10.1371/journal.pone.0221293)
Supplement: S1 File — (DOCX) [file pone.0221293.s015.docx]

**Supplemental methods**

**Statistical analyses for the primary study endpoint**

Uni- and multivariate Cox regression analyses were used to assess the effect of independent variables on the time to event for the study endpoint. Independent variables included those measured at the time of study enrolment (PLA_2_R1-ab levels, proteinuria, serum creatinine, age, sex, percentage of tubular atrophy and interstitial fibrosis, time between renal biopsy and study enrolment) and those measured during follow-up that represented certain events occurring in the course of the study (depletion of PLA_2_R1-ab, relapse of PLA_2_R1-ab positivity, remission of proteinuria, category of remission of proteinuria [partial remission versus complete remission], relapse of proteinuria, use of immunosuppressive treatment). The continuous independent variables PLA_2_R1-ab level, proteinuria, serum creatinine, percentage of tubular atrophy and interstitial fibrosis, time between renal biopsy and study enrolment were transformed to their binary logarithm prior to using them in Cox regression analyses. Time-dependent covariates were computed and used to test the proportional hazards assumption for individual independent variables. For independent variables measured at baseline we thus adjusted the analysis for potential time-varying effects during follow-up. For independent variables measured during follow-up we adjusted the effect of the variable for the time when its event (e. g. depletion or relapse of PLA_2_R1-ab, remission or relapse of proteinuria, start of immunosuppressive treatment) had occurred.

**Statistical analyses for depletion PLA_2_R1-ab, relapse of PLA_2_R1-ab, remission of proteinuria, and relapse of proteinuria**

We analysed the following parameters as potential predictors for depletion and relapse of PLA_2_R1-ab during follow-up: PLA_2_R1-ab levels, proteinuria, serum creatinine, age, gender, time between renal biopsy and study enrolment (all parameters collected at the time of study enrolment) and usage of immunosuppressive treatment during follow-up. The parameters analysed as potential predictors for remission of proteinuria during follow-up were collected: a) at the time of study enrolment (PLA_2_R1-ab levels, proteinuria, serum creatinine, age, gender, percentage of tubular atrophy and interstitial fibrosis, time between renal biopsy and study enrolment) and b) during follow-up (depletion of PLA_2_R1-ab, relapse of PLA_2_R1-ab, usage of immunosuppressive treatment). The same parameters were analysed as potential predictors for relapse of proteinuria during follow-up, in addition to the category of remission of proteinuria (partial remission versus complete remission). The analyses of PLA_2_R1-ab relapse and proteinuria relapse were restricted to those patients, who previously had had a remission of PLA_2_R1-ab and a remission of proteinuria, respectively. Moreover, the 21 patients who were tested negative for PLA_2_R1-ab by ELISA at baseline were not included in the analysis on depletion of PLA_2_R1-ab.

For all parameters collected at baseline we adjusted the analysis for potential time-dependent effects during follow-up, if the respective time-dependent term was significant. For all parameters collected during follow-up we always adjusted the predictive value of the variable for the time when the event had occurred (i.e. depletion or relapse of PLA_2_R1-ab, start of immunosuppressive treatment). In all these analyses we first performed a univariate Cox regression analysis including all independent variables. In a second step we ran a multivariable Cox regression analysis, in which we included and adjusted for all variables which had shown a statistically significant effect in the time-adjusted univariate analysis. Of these variables, only those significant after stepwise hierarchical-backward selection were retained in the final model. The continuous independent variables PLA_2_R1-ab levels, proteinuria, serum creatinine, percentage of tubular atrophy and interstitial fibrosis, time between renal biopsy and study enrolment were transformed to their binary logarithm prior to using them in cox regression analyses. Statistical significance was defined as p<0.05. All tests were two-tailed. Statistical analyses were performed using SPSS version 25.0 software.

**The following colleagues participated in the recruitment of the patients for this study (in alphabetical order)**

Aedtner F, Halberstadt, Ahmed A, Freiburg, Aign S, Berlin, Alscher D, Stuttgart, Altrogge H, Hamburg, Amir-Kabirian D, Hamburg, Arndt L, Buchholz, Arnold P, Siegen, Assenmacher A, Kamp-Lintfort, Baberg H, Berlin-Buch, Bachmann J, Arnsberg-Hüsten, Backs W, Hamburg, Bahte S, Stade, Bajeski V, Bielefeld, Baron J, Walsrode, Beck P, Langenfeld, Becker G, Becker S, Hamburg; Wattenscheid, Beckermann J, Vechta, Beckmann M-L, Bocholt, Beckmann S, Herford, Behrend M, Westerstede, Beimler J, Heidelberg, Bek M, Müllheim, Benz M, München, Benzing T, Köln, Beyer S, Hamburg, Bieringer M, Berlin-Buch, Biernat S, Varel, Biolik K, Berlin, Birkel J, Eggolsheim, Bischoff S, Dresden, Bödefeld T, Diepholz, Böhler J, Wiesbaden, Böhmer K P, Nürnberg, Bohling M, Wilhelmshaven, Bokemeyer D, Bochum, Bork T, Freiburg, Born B, Stade, Borst C, Neumünster, Boser M, Braunschweig, Bous A, Daun, Bozkurt F, Daun, Braasch E, Eberswalde, Bramstedt J, Bremerhaven, Braun J, Dingolfing, Breuer S, Kerpen, Brückner D, Dortmund, Brune K, Bad Rothenfelde, Bruns M, Hanau, Buchholz K, Emmendingen, Budde K, Berlin, Büldt E, Itzehoe, Burlage M, Hamburg, Burth E M, Paderborn, Busch M, Jena, Busch V, Münster, Büssemaker E, Hamm, Buth W, Minden, Clasen W, Münster, Claus M, Essen, Dannemann E-G, Gelsenkirchen, Daul A, Essen, David-Walek T, Kiel, Degenhardt S, Nettetal, Dellanna F, Düsseldorf, Dieckmann K, Flensburg, Dieterich F, Bad Oldesloe, Dillmann A, Bremen, Disteldorf E, Hamburg, Donauer J, Freiburg, Dorsch O, Kronach, Dose U, Siegburg, Döhler I, Dresden, Duvigneau D, Hamburg, Eckardt K-U, Erlangen, Edelmann B, Gelsenkirchen, Endreß H, Lörrach, Ernst M, Iserlohn, Fahrner, A, Freiburg, Falcke M, Hamburg, Feddersen A, Hamburg, Fellenberg N, Hamburg, Ferber J, Leverkusen, Feyerabend G, Reinbek, Fielitz J G, Elmshorn, Filejski W, Hamburg, Fina M, Hamburg, Fischer K-G, Lörrach, Floege J, Aachen, Fortnagel C, Pinneberg, Förster S, Ahrensburg, Frahm M, Eckernförde, Frank M, Pforzheim, Fricke L, Bochum, Fricke L, Lübeck, Friedrich B, Leonberg, Friedrichs I, Ansbach, Fuchs D, Oldenburg, Funk L, Freiburg, Gäckler D, Bochum, Galle J, Lüdenscheid, Ganzemüller C, Minden, Gerhardt T, Bonn, Gerke P, Lübeck, Gerth J, Zwickau, Glorius P, Templin, Gloy J, Hamburg, Gök Ö, Freiburg, Goldmann A, Neunkirchen, Gööck T, Arnstadt, Göttmann U, Mannheim, Graf G, Bad Bevensen, Gras K, Hamburg, Grewe B, Perleberg, Griesche-Philippi J, Lüneburg, Groll J, Berlin, Großer S, Hamburg, Grütter R, Dinslaken, Güz M A, Lüdenscheid, Haas C, Lübeck, Haberstroh U, Limburg, Hättig O, Offenburg, Hagenah F, Offenburg, Hahn K, Dortmund, Hamadeh A, Höxter, Hammerschmidt M, Erlangen, Heck M, Meppen, Heckel M, Kronach, Heering P J, Solingen, Hegner B, Berlin, Heidenreich S, Aachen, Heinrichs S, Düsseldorf, Hengemühle H, Bremen, Henrici E, Hamburg, Herb M, Lörrach, Hermann R, Müllheim, Hermle T, Freiburg, Hetzel G R, Düsseldorf, Heuschert J, Hamburg, Hintzen-Kruse C, Chemnitz, Hochtritt M S, Celle, Hohenstein B, Dresden, Hohmann M, Vechta, Holl W, Solingen, Hollenbeck M, Bottrop, Horn G, Erlangen, Hörnig B, Berlin, Hoyer J, Marburg/Lahn, Hugo C, Dresden, Hyndisurya A, Wien, Isbell L K, Freiburg, Jabs W, Berlin, Jacobson J, Dortmund, Jacobsen J, Winsen, Jäkel S, Bochum, Jank S, Erlangen, Jankrift P, Unna, Jasinski R, Freiburg, Jung O, Frankfurt a.M., Kaan T G, Leer, Kahlke D, Hamburg, Kämpf H, Sonneberg Kappey U, Hamburg, Keller F, Ulm, Ketteler M, Coburg, Kidder D, Buchholz, Kiziler F, Bochum, Kisner T, Köln, Klause N, Eckernförde, Klehr H U, Bonn, Kleinecke R, Bamberg, Klemm A, Jena, Knevels U, Unna, Knittel M, Witten, Koch C, Nürnberg, Koch M, Velbert, König D, Braunschweig, Köstler F, Elmshorn, Kohnle M, Hilden, Korschanowski A, Cuxhaven, Kortus-Götze B, Marburg/Lahn, Kositz H, Greiz, Krämer-Guth A, Freiburg, Kramer-Zucker A, Freiburg, Kreft B, Hildesheim, Kremerskothen R, Münster, Krenz I, Hamburg, Krumme B, Wiesbaden, Kube M, Hamburg, Kühns A, Hamburg, Kunigk F, Pinneberg, Kurschat C, Köln, Lange I, Perleberg, Lange D, Heilbad Heiligenstadt, Lange-Hüsken F, Hamburg, Laube C, Bremen, Laue O, Hannover-Langenhagen, Lauerwald W, Gera, Leidig B, Köln, Leimbach T, Berlin, Leingärtner T, Regensburg, Lepenies J, Berlin, Leschniewski M, Essen, Linden H M, Erfstedt, Linke B, Oldenburg in Holstein, Loke O, Lüdenscheid, Loley G, Osnabrück, Lohmann T, Dresden, Lorenz G, Melle, Lotz W P, Bremen, Loyen M, Münster, Lufft V, Rendsburg, Mander I, Lehrte, Marx C, Nordhausen, Meentzen C, Brake, Mees S, Hamburg, Meier-Sundhaußen G, Berlin-Marzahn, Meinke D, Lüdenscheid, Melching R, Gütersloh, Mertens P R, Magdeburg, Meßtorff K, Stade, Mettang T, Wiesbaden, Meyer E, Hamburg, Meyer W, Buchholz, Meyer-Delpho M, Siegburg, Middendorf C, Melle, Möller J, Leverkusen, Mohler C, Lüdenscheid, Morgenstern T, Koblenz, Müller R, Braunschweig, Müller R, Westerstede, Nesbigall T, Kaiserslautern, Neuber F, Zittau, Neumayer H-H, Berlin, Nöthen W, Siegburg, Oberle G, Hannover-Langenhagen, Oppermann M, Perleberg, Orlt A, Heilbad Heiligenstadt, Özcan F, Dortmund, Pape A, Walsrode, Pätzold C, Kamen, Pavenstädt H-J, Münster, Petersen M, Hamburg, Pfalzer B, Hamburg, Pilz M, Nottuln, Plöger A, Bielefeld, Plogmann C, Essen, Pöge U, Bonn, Potratz J, Rotenburg, Pützfeld C, Oberhausen, Regenberg M, Cuxhaven, Reichenberger F, Cottbus, Reinking R, Hamburg, Rensinghoff E, Wattenscheid, Ricken J, Rheine, Riechers G, Braunschweig, Riedasch M, Coesfeld, Rob P M, Lübeck, Roch P, Regensburg, Rockstroh S, Dresden, Röben T, Vechta, Rösch M A, Hamburg, Rosenburg C, Aschersleben, Rudnicki M, Innsbruck, Ruhberg B, Hamburg, Rump C, Düsseldorf, Sass C, Braunschweig, Schäfers R F, Oberhausen, Schaumann D, Hameln, Schenk L K, Münster, Schilken P, Paderborn, Schlee H, Weißenfels, Schmedding I, Freiburg, Schmidt R, Wesel, Schmiedel D, Bad Nenndorf, Schmidtmann K, Westerstede-Ocholt, Schmitz F-J, Minden, Schnegelsberg O, Winsen/Luhe, Schneidenbach R, Hamburg, Schneider A, Neuwied, Schneider J, Freiburg, Schneuzer C, Hamburg, Schnierda J, Waldshut-Tiengen, Schnitzler A, Lüneburg, Schreiber R, Coesfeld, Schröder S, Glauchau, Schüler A, Bad Nenndorf, Schüler M, Erlangen, Schulte-Vorwick M, Unna, Schulze M, Bad Zwischenahn, Schümann E, Riesa, Schwarz M, Verden, Schwertfeger E, Freiburg, Seifert A, Schleswig, Seifert A, Schleswig, Serwas A, Münster, Seuffert H-M, Karlsruhe, Seyfried J, Pforzheim, Siegmund J, Hamburg, Siwek-Orman, E, Arnstadt, Soldan E, Hamburg, Solf A, Heilbad Heiligenstadt, Sonneberg W P, Bad Rothenfelde, Spellmeyer G, Greven, Spieß M, Kaiserslautern, Stahn A, Hamburg, Steinhauer H B, Cottbus, Steinhoff J, Lübeck, Steinmetz M, Siegburg, Stiasny B, Schwabach, Storkenmaier R, Sindelfingen, Sturm E, Hamburg, Tacuri-Strasser D, Offenburg, Tegtmeier L, Bremen, Teichler S, Naumburg, Thiel M, Berlin, Thiele I, Reinbek, Tholl U, Kleve, Tiedeken P, Hamburg, Tillmann F-P, Emsdetten, Tjiang D, Königslutter, Toussaint, K, Hamburg, Treiber W, Neuwied, Tripps C, Oschersleben/OT Neindorf, Tröster S, Westerstede, Uhle A, Bochum, Unger T, Hamburg, Valentin R, Bielefeld, Vischedyk M, Paderborn, Vitu J, Hamburg, Volsek M, Essen, von Appen K, Hamburg, von Saß A Freiherr, Greiz, von Zitzewitz A, Ahrensburg, Voßkühler A, Bottrop, Wagner K, Hamburg, Walther T, Bad Kreuznach, Watter T, Freiburg, Weber M-H, Göttingen-Bovenden, Weidemann C, Erlangen, Weiner S, Trier, Weinert R, Bad Salzuflen, Weiß M, Hamburg, Weitzell R, Uelzen, Wiedemann F, Nagold, Wienbeck C, Hamburg, Wiesener M, Erlangen, Willecke J, Freiburg, Wilms H, Hamburg, Wirtz G, Kamen, Wittberger W, Gladbeck, Witthus M, Köln, Wittmaack D, Itzehoe, Woggan J, Hamburg, Wolf G, Jena, Wolfram L, Hamburg, Wollweber T, Wadersloh, Wullstein H-G, Hamburg, Zander A, Verden, Zeier M, Heidelberg
